# TREX tetramer disruption alters RNA processing necessary for corticogenesis in THOC6 Intellectual Disability Syndrome

**DOI:** 10.1038/s41467-024-45948-y

**Published:** 2024-02-22

**Authors:** Elizabeth A. Werren, Geneva R. LaForce, Anshika Srivastava, Delia R. Perillo, Shaokun Li, Katherine Johnson, Safa Baris, Brandon Berger, Samantha L. Regan, Christian D. Pfennig, Sonja de Munnik, Rolph Pfundt, Malavika Hebbar, Raúl Jimenez-Heredia, Elif Karakoc-Aydiner, Ahmet Ozen, Jasmin Dmytrus, Ana Krolo, Ken Corning, E. J. Prijoles, Raymond J. Louie, Robert Roger Lebel, Thuy-Linh Le, Jeanne Amiel, Christopher T. Gordon, Kaan Boztug, Katta M. Girisha, Anju Shukla, Stephanie L. Bielas, Ashleigh E. Schaffer

**Affiliations:** 1 Department of Human Genetics, University of Michigan Medical School, Ann Arbor, MI 48109 USA; 2 Advanced Precision Medicine Laboratory, The Jackson Laboratory for Genomic Medicine, Farmington, CT 06032 USA; 3 Department of Genetics and Genome Sciences, Case Western Reserve University School of Medicine, Cleveland, OH 44106 USA; 4 Department of Medical Genetics, Sanjay Gandhi Postgraduate Institute of Medical Sciences, Lucknow, Uttar Pradesh 226014 India; 5 Division of Pediatric Allergy and Immunology, School of Medicine, Marmara University, Istanbul Jeffrey Modell Diagnostic and Research Center for Primary Immunodeficiencies, The Isil Berat Barlan Center for Translational Medicine, Istanbul, 34722 Turkey; 6 Department of Human Genetics, Radboud University Medical Centre Nijmegen, Nijmegen, 6524 the Netherlands; 7 Division of Genetic Medicine, Department of Pediatrics, University of Washington, 98195 Seattle, WA, USA; 8 Ludwig Boltzmann Institute for Rare and Undiagnosed Diseases, Vienna, 1090 Austria; 9 Research Centre for Molecular Medicine of the Austrian Academy of Sciences, Vienna, 1090 Austria; 10 Greenwood Genetic Center, Greenwood, SC 29646 USA; 11 Section of Medical Genetics, SUNY Upstate Medical University, Syracuse, NY 13210 USA; 12 Imagine Institute, INSERM U1163, Paris Cité University, Paris, 75015 France; 13 Service de Médecine Génomique des Maladies Rares, Hôpital Necker-Enfants Malades, AP-HP, Paris, 75015 France; 14 Department of Pediatrics and Adolescent Medicine, Medical University of Vienna, Vienna, 1090 Austria; 15 St. Anna Children’s Hospital and Children’s Cancer Research Institute, Department of Pediatrics, Medical University of Vienna, Vienna, 1090 Austria; 16 Department of Medical Genetics, Kasturba Medical College, Manipal, Manipal Academy of Higher Education, Manipal, 576104 India; 17 Department of Pediatrics, University of Michigan Medical School, Ann Arbor, MI 48109 USA

**Keywords:** Disease model, RNA splicing

## Abstract

*THOC6* variants are the genetic basis of autosomal recessive *THOC6* Intellectual Disability Syndrome (TIDS). THOC6 is critical for mammalian Transcription Export complex (TREX) tetramer formation, which is composed of four six-subunit THO monomers. The TREX tetramer facilitates mammalian RNA processing, in addition to the nuclear mRNA export functions of the TREX dimer conserved through yeast. Human and mouse TIDS model systems revealed novel THOC6-dependent, species-specific TREX tetramer functions. Germline biallelic *Thoc6* loss-of-function (LOF) variants result in mouse embryonic lethality. Biallelic *THOC6* LOF variants reduce the binding affinity of ALYREF to THOC5 without affecting the protein expression of TREX members, implicating impaired TREX tetramer formation. Defects in RNA nuclear export functions were not detected in biallelic *THOC6* LOF human neural cells. Instead, mis-splicing was detected in human and mouse neural tissue, revealing novel THOC6-mediated TREX coordination of mRNA processing. We demonstrate that THOC6 is required for key signaling pathways known to regulate the transition from proliferative to neurogenic divisions during human corticogenesis. Together, these findings implicate altered RNA processing in the developmental biology of TIDS neuropathology.

## Abstract (web summary)

THOC6 is required for TREX tetramer formation. Analysis of pathogenic THOC6 variants differentiate the conserved mRNA export functions of TREX dimers and RNA processing functions of TREX tetramers underlying THOC6 Intellectual Disability Syndrome.

## Introduction

Intellectual disability (ID) is a clinical feature of neurodevelopmental disorders characterized by limitations in cognitive ability and adaptive behavior^1^. Broad use of exome-based genetic testing in recent years has greatly increased the list of genes implicated in syndromic ID^2–6^. Monogenetic etiologies are a major basis of syndromic ID, with many following an autosomal-recessive mode of inheritance^7^. Beaulieu-Boycott-Innes Syndrome (MIM# 613680), also known as THOC6 Intellectual Disability Syndrome (TIDS)^8^, is one such recessive ID due to biallelic *THOC6* variants^9–19^. Individuals with TIDS exhibit moderate to severe syndromic ID with microcephaly, developmental delay, and multi-organ involvement^8^.

THOC6 is a subunit of the six member THO (suppressors of the Transcriptional defects of Hpr1Δ by Overexpression) complex, which serves as a core component of the transcription/export (TREX) complex^20^. TREX is critical for licensing export factors required for nuclear pore docking and export of mRNA from the nucleus to the cytoplasm^21–24^. While this is a conserved function of TREX, there are notable species differences in TREX composition that mirror the evolutionary increase of RNA processing complexity. In budding yeast, TREX is a dimer composed of two conserved five-subunit THO monomers, of which THOC6 is the notable exception. TREX monomers dimerize via the coiled-coil domains of Thp2 and Mft1, the budding yeast orthologs of THOC5 and THOC7^25^. In mammals, TREX is a tetramer, where dimers of THO monomers are tethered by THOC6^25^. Increased size and molecular complexity of the mammalian TREX tetramer correlates with increased RNA processing demands that have evolved in organisms with higher transcriptome complexity and messenger ribonucleoprotein complex (mRNP) composition, namely expression of long genes with high levels of complex splicing patterns^26^. Notably, in yeast, introns constitute 5% of yeast genes, are comparably short, and are limited to one per gene^27–30^. By contrast, introns comprise ~24% of mammalian genomes^31^ and are present in >95% of all human genes^27,28,32^. This suggests that THOC6 has evolved to permit the formation of larger TREX complexes, which has implications for the coordination of RNA processing in metazoans relative to budding yeast^25,33^.

TREX complex-mediated mRNP nuclear export is an evolutionarily conserved function^34^. Consistent with this essential function, genes that encode TREX dimer components *THOC1*, *THOC3*, *THOC5*, and *THOC7* exhibit a high probability of intolerance (pLI) to loss-of-function (LOF) variants in gnomAD and have not been identified as the genetic basis of developmental disorders, raising the possibility that genetic disruption of these THO components is embryonic lethal in humans. *THOC2*, a THO monomer component, is the genetic basis of an X-linked neurodevelopmental disorder that does not phenocopy TIDS^35^. Functionally, depletion of THO components THOC1-THOC5 and THOC7 leads to significant nuclear export defects^36^. These findings confirm the conservation of TREX mRNA export functions, but do not discriminate between the requirement for dimer versus tetramer formation for this function.

Unlike dimers, the TREX tetramer recruits functionally diverse auxiliary factors that lack orthologs in budding yeast. DDX39A, CHTOP, UIF, LUZP4, POLDIP3, ZC3H11A, ERH, ZC3H18, SRRT, and NCBP3 complex with tetrameric TREX and participate in mRNP processing and export, which includes mRNA 5’ capping, splicing, and 3’ end processing to create mRNPs capable of translocating through the nuclear pore complex into the cytoplasm^22,34,37^. Increased size of the TREX-tetramer complex may represent greater RNA processing functionality as the complexity of mRNP processing evolved^34,38^. Likewise, the larger complex would enable TREX to serve as an RNA chaperone to prevent the formation of DNA-RNA hybrid or R-loop structures, with the emergence of longer transcripts that undergo extensive splicing, that can promote genome instability^39,40^. This idea is supported by the genetics associated with conserved TREX auxiliary factor ALYREF, whose ortholog Yra1 is essential for viability in yeast, yet not in metazoans^41,42^. *ALYREF* is the genetic basis of congenital disease in humans^43^, suggesting evolved redundancy in TREX tetramer functions in metazoans^44–47^.

In this study we used human genetics to expand the number of reported *THOC6* variants and the phenotypic spectrum of TIDS. We showed that pathogenic *THOC6* nonsense and missense variants function through a LOF genetic mechanism. We demonstrated that TREX tetramer auxiliary protein ALYREF immunoprecipitated with TREX complex components in control cells, but not *THOC6* LOF iPSCs, in line with altered TREX tetramer formation but not TREX dimers. This distinction allowed THOC6-dependent tetramer function(s) to be evaluated, relative to dimer function(s). We generated mouse (in vivo*)* and human (in vitro) models of neural development to investigate shared pathogenic mechanisms of mammalian *THOC6* LOF. Biallelic *Thoc6* LOF mice are embryonic lethal, which fails to phenocopy TIDS and implicates species differences. In *THOC6* LOF models, global mRNA nuclear export occurred similar to control NPCs, suggesting export is a conserved TREX-dimer function. RNA sequencing of NPCs revealed mRNP processing defects in both mouse and human *THOC6* LOF cells. We showed that selective mRNA processing in *THOC6* LOF organoids results in dysregulation of NPC proliferation and excitatory neuron differentiation. Our findings reveal a broader supportive role for the TREX tetramer across RNA processing than has previously been attributed to THO/TREX.

## Results

### ***THOC6*variants are the genetic basis of TIDS**

**Fig. 1:**
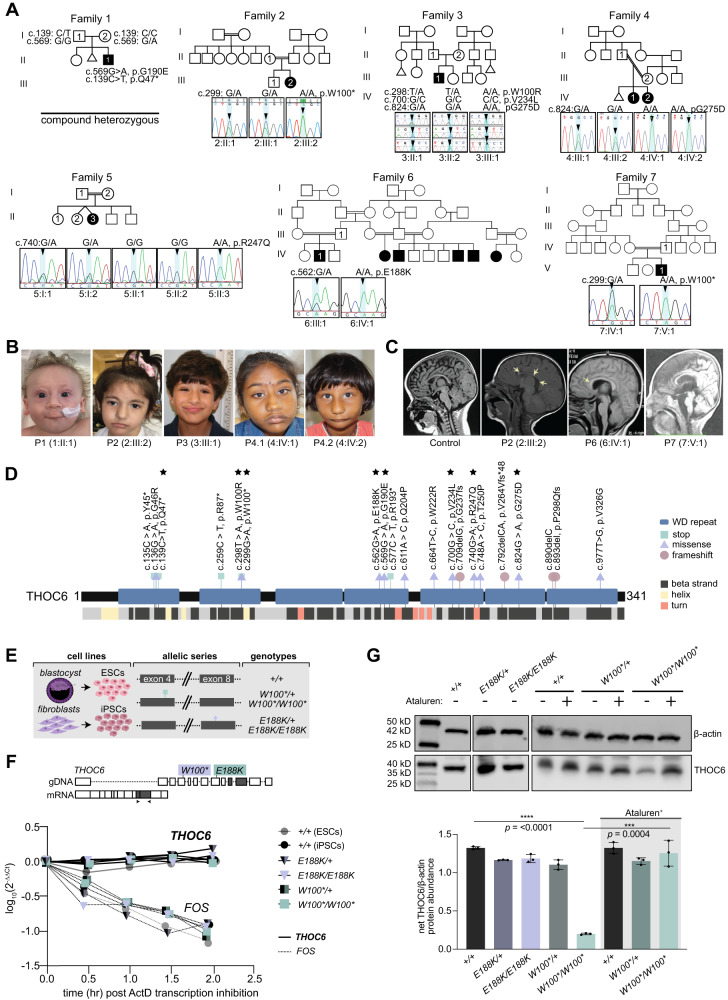
Biallelic pathogenic variants in *THOC6* cause syndromic intellectual disability. **A** Pedigree drawings of segregating TIDS phenotypes in families 1–7, with generations listed on the left-hand side. Females are represented as circles and males are denoted by squares. Miscarriages are denoted by small triangles. Affected family members are indicated by solid black coloring while unaffected are unfilled. Consanguineous partnerships are represented by double lines. Chromatograms from Sanger sequencing confirmation of THOC6 (NM_024339.5) genotypes are provided for each tested family member in families 1–7. **B** Facial photographs of P1-P4. **C** Sagittal brain MRI showing corpus callosum dysgenesis (P2, P6, & P7) and cortical and cerebellar atrophy (P6) compared to control (left). **D** Canonical THOC6 protein map consisting of 341 amino acids. WD40 repeat domains 1–7 are denoted by purple rectangles. The location of known pathogenic variants is annotated relative to the linear protein map (top) and secondary structure (below). Variants reported in the present study are distinguished by a black star. Missense (blue triangle), nonsense (red square), and frameshift (green circle). **E** Schematic of patient and control-derived human cell types and respective genotypes. **F** Levels of *THOC6* mRNA (solid line) following ActD transcriptional inhibition compared to *FOS* mRNA (dotted line) in human ESC/iPSCs across genotypes*: THOC6*^*+/+*^ ESCs (grey solid circle), *THOC6*^*+/+*^ iPSCs (black solid circle), *THOC6*^*E188K/+*^ (purple-black triangle), *THOC6*^*E188K/E188K*^ (solid light purple triangle), *THOC6*^W100*/+^ (green-black square), and *THOC6*^W100/W100*^ (light green square). Values calculated relative to *GAPDH* reference mRNA. This experiment was repeated three independent times with similar results. **G** Western blot of human ESC/iPSCs indicating reduced THOC6 protein expression in *THOC6*^W100*/W100*^ iPSCs compared to unaffected controls. Enhanced expression of THOC6^W100*^ by Ataluren treatment (30 μM). Abundance quantifications relative to β-actin control (right). *N* = 3 independent abundance measurements per genotype. Genotypes*: THOC6*^*+/+*^ iPSCs (black), *THOC6*^*E188K/+*^ (dark purple), *THOC6*^*E188K/E188K*^ (light purple), *THOC6*^W100*/+^ (dark green), and *THOC6*^W100/W100*^ (light green). Data are represented as mean ± SEM. Significance measured by two-tailed, unpaired *t* test. **** indicates *p*-value = <0.0001. (For anti-THOC6 antibody validation, see Fig. S2A). This experiment was repeated two independent times with similar results. Source data are provided as a Source Data file.

While initially detected in Hutterite populations^12^, a growing number of pathogenic biallelic *THOC6* variants are being discovered across the globe in individuals of diverse ancestry^9–11,13–19,48^. As part of an ongoing effort to determine the genetic etiologies of syndromic ID, we identified nine *THOC6* variants by exome-based genetic testing (Fig. 1A). Six variants were found to alter amino acids p.W100, p.G190, p.V234, and p.G275, previously associated with TIDS, whereas three *THOC6* alleles (c.139 C > T, p.Q47*; c. c.562 G > A, p.E188K; c.740 G > A, p.R247Q) were novel. These newly described cases exhibit clinical features of TIDS, namely global developmental delay, moderate to severe ID and facial dysmorphisms (Fig. 1B). Cardiac and renal malformations, structural brain abnormalities with and without seizures, urogenital defects, recurrent infections, and feeding complications were also detected with variable expressivity (Fig. 1C). The multiorgan involvement of this developmental syndrome is further highlighted by detailed clinical summaries for affected individuals provided in Table S1.

The novel *THOC6* variants are comparable to previously described nonsense and missense variants that contribute equally to the severity of TIDS phenotypes. We detected a nonsense *THOC6* c.139 C > T, p.Q47* variant in exon 2 of P1, a missense c.740 G > A, p.R247Q variant in exon 11 of P5, and a missense c.562 G > A, p.E188K variant in P6 (Fig. 1A, C). THOC6 is comprised of seven WD40 repeat domains (Fig. 1D) that form a β-propeller structure when folded. These novel variants, like other clinically relevant *THOC6* variants, map to the WD40 repeats of THOC6 (Fig. 1D). Among all *THOC6* cases presented here, and in the literature, there is no genotype-phenotype correlation across variants, which supports a LOF pathogenic mechanism for nonsense and missense *THOC6* variants.

### ***THOC6*variants act through loss-of-function genetic mechanism**

To investigate the molecular pathology of *THOC6* nonsense and missense variants, we used embryonic stem cells (ESCs) and iPSC lines to assess *THOC6* mRNA stability (Fig. 1E). iPSCs were reprogrammed from two individuals with TIDS (6:IV:1 (P6)*, THOC6*^*E188K/E188K*^ and 7:V:1 (P7), *THOC6*^W100*/W100*^) and their respective unaffected heterozygous parent (6:IV:2, *THOC6*^*E188K/+*^ and 7:IV:1, *THOC6* ^W100*/+^) (Fig. 1E), preserving the shared genetic background between affected and unaffected conditions. Genotypes were confirmed routinely during culturing by Sanger Sequencing (Fig. S1A and B). To assess mRNA transcript stability, ESCs/iPSCs were treated with Actinomycin D for 2-8 hours, to inhibit nascent transcript production over the time course of RNA collection. The stability of *THOC6* mRNA transcripts was assessed by RT-qPCR and compared to unstable mRNA (*FOS*) that is quickly degraded (Figs. 1F and S1C–S1E)^49^. We predicted that nonsense pathogenic variants destabilize *THOC6* mRNA and thereby make them vulnerable to mRNA decay, like that observed for *FOS*. *THOC6* mRNA transcripts remained stable between genotypes, with the c.299 G > A, p.W100* and c.562 G > A, p.E188K variants exhibiting similar stability to wildtype transcripts (Figs. 1F and S1C–E)^49^.

*THOC6* missense variants produce stable *THOC6* mRNA, and missense THOC6 protein is expressed at a similar level to *THOC6*^*+/+*^ controls in *THOC6*^*E188K/E188K*^ iPSCs (Fig. 1G). This finding led us to predict that missense THOC6 protein activity is disrupted relative to wildtype THOC6 protein functions. We also observed that nonsense *THOC6* variants produce highly stable *THOC6* transcripts from the *THOC6*^W100*^ nonsense allele. Nonsense-carrying transcripts are predicted to undergo nonsense-mediated decay and ablate protein expression. Unexpectedly, a minimal but measurable quantity of THOC6 protein is observed in *THOC6*^W100*/W100*^ iPSCs by Western blot, whereas THOC6 expression in *THOC6*^W100*/+^ iPSCs is comparable to *THOC6*^*+/+*^ controls (Fig. 1G). To determine if suppression of translation termination, also referred to as readthrough, could contribute to a broader mechanism that permits this sparse expression from the *THOC6*^W100*^ allele, cells were treated with 30 μM Ataluren. This compound induces protein synthesis through premature termination codons, often producing full-length missense protein. Increased protein abundance of THOC6 was observed in Ataluren-treated *THOC6*^W100*/W100*^ iPSCs, suggesting that rare translation from the *THOC6* ^W100*^ mRNA may occur, but is predicted to carry nonsynonymous substitutions (Fig. 1G). Resultant missense proteins may generate pathogenic functional consequences. This finding is appreciable in Ataluren-treated *THOC6*^W100*/W100*^ iPSCs by Western blot but does not produce a significant change in THOC6 levels in Ataluren-treated *THOC6*^W100*/+^ iPSCs. This may be due to control-level THOC6 expression that is measured in *THOC6*^W100*/+^ iPSCs. Truncated THOC6 protein was not observed in either *THOC6*^W100*/W100*^ nor *THOC6*^W100*/+^ iPSCs by Western blot analysis, and THOC6 protein abundance in *THOC6*^*E188K/E188K*^ iPSCs was comparable to wildtype. The clinical overlap between these pathogenic alleles suggests that the resultant variant THOC6 is likely functionally inactive.

### ***THOC6*variants disrupt the TREX tetramer**

**Fig. 2:**
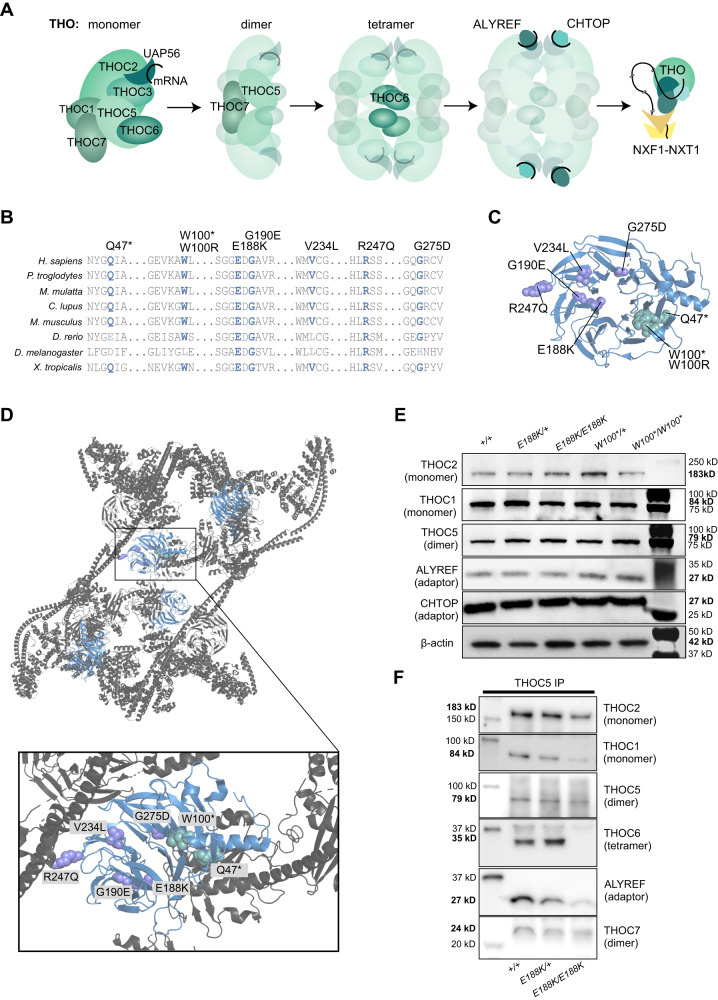
Genetic mechanism of biallelic pathogenic *THOC6* variants. **A** Schematic of TREX core tetrameric assembly mediated by THOC6 with functional implications for mRNA processing and export based on the published crystal TREX structure^25^. **B** Amino acid alignment showing conservation of affected residues (blue font) for pathogenic variants in present clinical study. Variants mapped to **C** THOC6 folded β-propeller structure and **D** THO/TREX complex. Missense variant residues are represented with purple cartoon spheres; nonsense variant residues are represented with green cartoon spheres. **E** Steady-state protein abundance for THO/TREX complex members. This experiment was independently repeated four times with similar results. **F** THOC6 and ALYREF abundance following co-immunoprecipitation with THOC5 for unaffected control, unaffected missense heterozygous, and affected missense homozygous iPSCs. This experiment was independently repeated three times with similar results. Source data are provided as a Source Data file.

Based on the crystal structure of TREX^25^, THOC6 tethers TREX dimers to form a tetramer that permits interaction with axillary molecules, such as ALYREF and CHTOP. THO monomers are dimerized by THOC5 and THOC7. THOC6 is positioned at the TREX core interface where it interacts with THOC5 to facilitate tetramerization (Fig. 2A). Interactions with THOC5 are mediated through the WD40 domains of THOC6. Not all pathogenic *THOC6* variants alter residues conserved across metazoan species (Fig. 2B). This pattern of conservation mirrors the evolving function of THOC6 in TREX and its composition variability. The allelic series of *THOC6* variants implicate a pathogenic mechanism whereby lack of THOC6, or expression of variant THOC6, prevents tetramer formation while leaving TREX dimers intact (Fig. 2C, D). Consistent with this model, protein expression of THO dimer components was comparable to wildtype controls in *THOC6* affected cells by Western blot (Fig. 2E). Likewise, THO dimer proteins localized to nuclear speckle compartments that exhibited the same nuclear configuration in affected cells by immunohistochemistry (Fig. S1F and G).

As prior work has demonstrated that THOC6 directly heterodimerizes and homodimerizes with THOC5 and THOC6, respectively, to facilitate tetramer formation^25,33^, we next wanted to assess the ability of THOC6 missense p.E188K to associate with TREX. We performed co-immunoprecipitation of THOC5 from THOC6 p.E188K affected and control cells and found a complete loss of THOC5-THOC6 interaction, demonstrating the missense variant disrupts the ability of THOC6 to bind to THOC5 (Figs. 2F and S1H). THOC1 and THOC2 were associated with THOC5 at comparable levels between affected and control conditions (Figs. 2F and S2B), indicating that the TREX complex was stable in the presence of THOC6 variants, but likely in a dimer conformation. We also examined the association of the adaptor protein ALYREF, which binds mRNA during co-transcriptional processing and export, with the THO subcomplex and found the interaction was reduced in affected cells (Figs. 2F and S1H). Together, these findings suggest a THOC6-dependent association of ALYREF to THO, with implications for the affinity of other adaptors due to the potential disruption of TREX tetramer formation.

### **Global mRNA export is unchanged by*THOC6*variants**

**Fig. 3:**
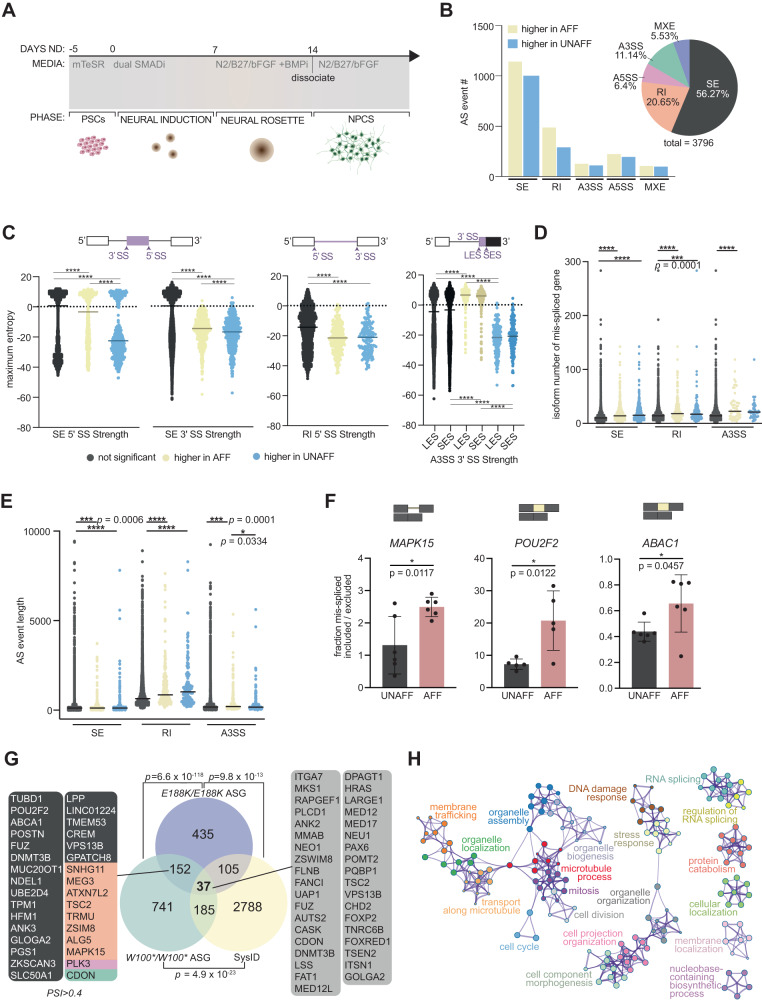
Characterization of alternative splicing events in *THOC6* affected hNPCs. **A** Differentiation protocol to derive human neural progenitor cells from affected and unaffected ESC/iPSCs. **B** Combined rMATS summary results of statistically significant AS events in *THOC6*^W100*/W100*^ and *THOC6*^*E188K/E188K*^ hNPCs relative to *THOC6*^W100*/+^ control hNPCs. Event type (pie chart) and inclusion status (bar chart). Yellow, higher inclusion in affected condition. Blue, higher inclusion in unaffected conditions. **C** Significant splice site strength score differences at mis-spliced events in affected hNPCs based on the maximum entropy model. Black, non-significant events. Yellow, higher inclusion in affected condition. Blue, higher inclusion in unaffected conditions. Significance obtained by two-tailed, unpaired *t* test. **** indicates *p* = <0.0001. Transcript number per AS gene **D** and AS event length **E** in *THOC6*^W100*/W100^ and *THOC6*^*E188K/E188K*^ vs. *THOC6*^W100*/+^ NPCs. Black, non-significant events. Yellow, higher inclusion in affected condition. Blue, higher inclusion in unaffected conditions. Significance obtained by two-tailed, unpaired *t* test. **** indicates *p* = <0.0001. **F** RT-PCR AS validations of SE (*ABAC1*, *POU2F2*) and RI (*MAPK15*) events in *n* = 3 additional biological replicates of hNPCs per genotype (6 per condition) with quantified mis-spliced ratios. Significant outliers were identified for *POU2F2* and were then excluded from analysis. Data shown as mean ± SEM. Significance obtained by two-tailed, unpaired *t* test. UNAFF, unaffected heterozygous control (black); AFF, homozygous affected (red). **G** Venn diagram of overlap of *THOC6* ^W100*/W100*^ and *THOC6*^*E188K/E188K*^ AS genes and all syndromic intellectual disability genes included in the SysID database. Overlap significance tested by one-sided Fisher’s exact test. ASG, alternatively spliced genes. **H** Metascape analysis on combined significant mis-spliced events (FDR < 0.05) in *THOC6*^*E188K/E188K*^ and *THOC6*^W100*/W100*^ NPCs.

mRNA export is an important TREX function. This TREX function was investigated to functionally assess the pathogenicity of *THOC6* variants in human neural progenitor cells (hNPCs) differentiated from iPSCs (Fig. 3A). Primary microcephaly is a clinical feature of TIDS, attributed to developmental defects in hNPCs during corticogenesis, making this an important cell type to investigate THOC6 pathogenesis. Oligo-dT fluorescent in situ hybridization (FISH) was performed on hNPCs to visualize poly **A** + RNA signal in nuclear and cytoplasmic cellular fractions (Fig. S2B–D). Defects in RNA export result in the accumulation of polyadenylated (poly **A** + ) RNA in the nucleus, with enrichment at nuclear speckle, as is observed in cells treated with wheat germ agglutinin (WGA), a potent inhibitor of all nuclear pore transport and the positive control for export defects^50,51^ (Fig. S2B). Despite the role of TREX in RNA export, the nuclear-to-cytoplasmic (N/C) poly **A**+ fluorescent intensity ratio was not increased in *THOC6* affected cells. No statistical difference in the (N/C) poly **A**+ fluorescent intensity ratio was identified between*THOC6* affected and unaffected control hNPCs, while a highly significant increase in the N/C poly **A**+ signal intensity ratio was measured in *THOC6*^*+/+*^ hNPCs treated with WGA attributed to high accumulation of poly **A** + RNA in the nucleus (Fig. S2C)^50^. The absence of bulk poly **A** + RNA export defect in *THOC6*-affected hNPCs suggests the TREX tetramer is not required for this molecular function (Fig. S2D) and may instead impinge on mRNP processing functions upstream of export.

### **Alternative splicing is disrupted in*THOC6*LOF hNPCs**

RNA export is an important step in the mRNP life cycle. However, TREX has documented functions across the broader processes of mRNA biogenesis^34,36,52–54^. TREX is recruited to the 5’ end of maturing mRNPs during transcription and associates with critical splicing factors that function in co-transcriptional mRNA processing^52^. To determine if mRNA processing is vulnerable to THOC6 LOF TREX defects, splicing and expression were assessed. RNA-sequencing (RNAseq) of ribosomal (r)RNA-depleted control, unaffected heterozygous, and homozygous affected hNPCs samples was performed (Supplementary Data 1). Principal components analysis (PCA) demonstrates reproducibility between replicates and genotypic differences (Fig. S3A). Comparative splicing analysis was carried out using the rMATS pipeline on biallelic *THOC6*^*E188K/E188K*^ and *THOC6*^W100*/W100*^ samples versus respective heterozygous controls (Supplementary Data 2)^55^. A total of 3796 alternative splicing (AS) events were significantly enriched in affected NPCs (Fig. 3B). The major AS events were categorized: skipped/cassette exon (SE), alternative 5’ splice site (A5SS), alternative 3’ splice site (A3SS), retained intron (RI), and mutually exclusive exon (MXE). SE (56%, 2136 of 3796) and RI (21%, 784 of 3796) were both overrepresented AS events in affected samples. This high frequency of RIs detected in *THOC6* LOF is a notable outlier compared to observed ratios of AS events associated with other splicing factors (Fig. 3B)^56–61^. Likewise, the A3’SS and A5’SS events exhibit a slight trend towards inclusion, consistent with the high frequency of RIs (Fig. 3B). SE and RI splicing events occur by distinct molecular mechanisms, mediated by exon junction complex (EJC) pathways. Detection of defects in both splicing categories suggests that the TREX tetramer serves as a multifunctional molecular platform for coordinating complex splicing events, as opposed to regulation of a specific subset of splicing events controlled by association with and function of individual RNA splicing factors. In agreement with this finding, we did not find a consistent, enriched motif for specific RNA-binding proteins at differentially spliced junctions as assessed by CentriMo analysis. These findings highlight an important role for THOC6-dependent TREX splicing in mRNA processing in hNPCs.

Differential expression was quantified from hNPC RNAseq data to identify how the ratio of THOC6-affected AS events correlates to transcriptomic changes. Transcription factor analysis (GSEA and ChEA3 transcription factor target gene analysis) did not reveal trans-regulatory elements responsible for the THOC6-affected differential splicing pattern, positing that cis-elements may underlie these differences. A maximum entropy model that assesses short sequence motif distributions was used to test the strength of the donor (5’) and acceptor (3’) of AS events^62^. A general trend towards weaker splice sites were detected at differential SE, RI, and A3SS events in affected cells (unpaired two-tailed *t* test, Fig. 3C). The SE, RI, and A3SS events were enriched in genes with a disproportionately high number of isoforms that show dependence on weak, alternative/cryptic splice sites to facilitate isoform diversity (Fig. 3D)^63^. RI events in affected hNPCs also had weaker splice sites compared to controls, suggesting that THOC6 deficiency induces mis-splicing at weak splice sites. In addition, the AS SE, RI, and A3SS events in affected *THOC6* hNPCs impacted exons/introns that are significantly longer than nonsignificant events (Fig. 3E). Likewise, the length of introns retained in RI events were significantly longer, with a 1.4-fold increase in length quantified for significant RI events (*p* = <0.0001, unpaired two-tailed *t* test, Fig. 3E). Lastly, no positional bias was observed for AS events (Fig. S3D and S3F). To validate our bioinformatic analysis, AS inclusion trends in select top, shared events were validated by RT-qPCR (unpaired two-tailed *t* test, Fig. 3F). Together, the RNA misprocessing signature across diverse SE and RI events at weak splices sites suggests impaired splicing fidelity from depletion of THOC6.

To investigate the role of this RNA misprocessing in ID pathology, THOC6-affected AS events were intersected with the genes that are known to cause syndromic ID, deposited in the SysID database (SysIDdb). A total of 152 genes with significant AS events included or excluded in >10% of transcripts in hNPCS were detected in nonsense and missense affected genotypes (Fig. 3G). A total of 185 AS genes in *THOC6*^W100*/W100*^ and 105 AS genes in *THOC6*^*E188K/E188K*^ hNPCs are known genes causative for syndromic ID represented in the SysIDdb (Fig. 3G). Aberrantly spliced ID genes were identified in both THOC6-affected genotypes, consistent with a role for THOC6 in ID. Thirty-seven ID genes (1.3% of SysIDdb) are AS in both affected genotypes, identifying genes for shared mechanisms that may preferentially contribute to TIDS pathology. To identify biological mechanisms implicated by THOC6-dependent AS, biological pathway enrichment analysis was performed on mis-spliced genes in affected cells. Genes with differential splicing were significantly enriched for functions in RNA splicing, cell projection organization, membrane trafficking, organelle organization, mitosis cell cycle, and DNA damage response (Fig. 3H). RNA processing is tightly controlled by feedback loops (e.g., auto-repression by poison exons or intron retention), which would explain how effects on cis-elements may lead to changes in *trans* factors (i.e., AS events in splicing regulatory factors).

### ***THOC6*LOF hNPC splicing defects dysregulate cortical differentiation pathways**

**Fig. 4:**
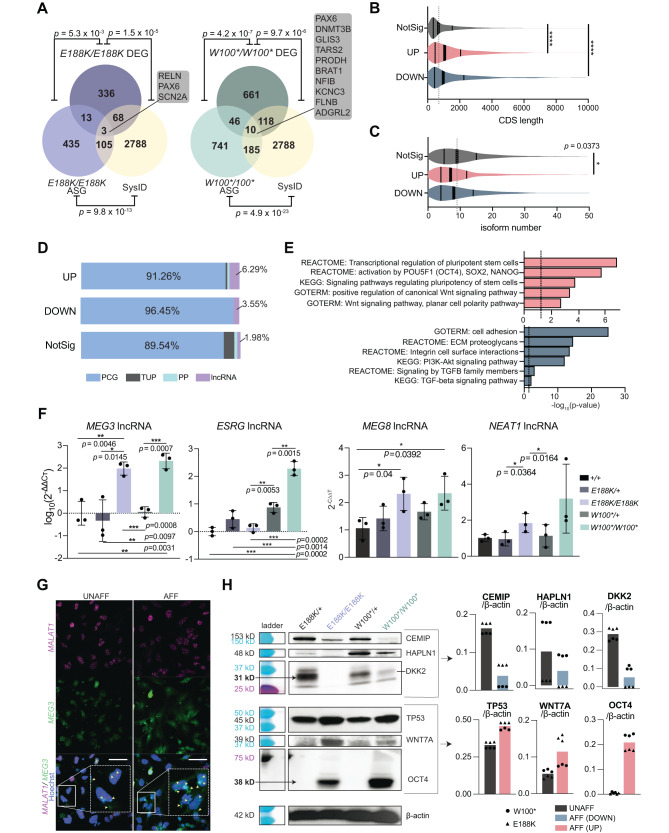
Differential expression analysis in affected hNPCs. **A** Venn diagram of gene overlap of *THOC6* ^W100*/W100*^ and *THOC6*^*E188K/E188K*^ affected genes and all syndromic intellectual disability genes included in the SysID database. Overlap significance tested by one-sided Fisher’s exact test. DEG, differentially expressed genes; ASG, alternatively spliced genes. **B** Violin plot of coding sequence (CDS) length of combined DEGs in affected cells compared to non-significant genes. NotSig, non-significant genes (dark grey); UP, upregulated (coral); DOWN, downregulated (dark blue). Significance, two-tailed unpaired *t* test, *p* = <0.0001 (****). **C** Violin plot of isoform number of combined DEGs in affected cells compared to non-significant genes. NotSig, non-significant genes (dark grey); UP, upregulated (coral); DOWN, downregulated (dark blue). Significance, two-tailed unpaired *t* test, *p* = 0.0373. **D** Percentage of gene type by condition for combined DEGs in affected hNPCs. NotSig, non-significant genes; UP, upregulated. DOWN, downregulated; lncRNA, long non-coding RNA (lavender); PP, processed pseudogene (turquoise); TUP, transcribed unprocessed pseudogene (grey); PCG, protein-coding gene (blue). **E** DAVID biological pathway enrichment analysis of combined upregulated genes (top, red) and downregulated genes (bottom, blue) in *THOC6* affected hNPCs. **F** qPCR relative abundance quantifications (2^-ΔΔCt^) for *MEG3*, *ESRG*, *MEG8*, and *NEAT1* in hNPCs*. n* = 3 experimental replicates of 3 biological replicates per genotype. Genotypes: *THOC6*^*+/+*^ (black), *THOC6*^*E188K/+*^ (dark purple), *THOC6*^*E188K/E188K*^ (purple), *THOC6*^W100*/+^ (dark green), *THOC6*^W100*/W100*^ (green). Data shown as mean ± SEM. Significance, two-tailed unpaired *t* test. This experiment was repeated twice independently and showed similar results. **G** RNA FISH probing for *MEG3* and *MALAT1* in affected and unaffected hNPCs. Cell inset showing *MEG3* expression and localization differences with yellow arrows in merged image. Scale bar 50 μm. UNAFF, unaffected (*THOC6*^W100*/+^); AFF, affected (*THOC6*^W100*/W100*^) **H** Representative Western blot images (left) and protein abundance quantification (normalized to B-actin control) for validation of top downregulated (blue) and upregulated (coral) genes across genotypes. Triangles specify genotypes with E188K variant, and circles specify the W100* variant. *N* = 3 technical replicates per genotype (*n* = 6 total replicates per condition). This experiment was performed once. Source data are provided as a Source Data file.

Pathogenic THOC6 mRNA processing events, such as alternative SE and RI splicing, impact gene expression through multiple mechanisms, including changing the ratio of expressed isoforms and destabilizing mRNAs by intron inclusion. AS events were correlated to differential expression of THOC6 homozygous affected and heterozygous unaffected controls (Figs. 4A, S4A, and B). Among the 336 differentially expressed genes (DEGs) in *THOC6*^*E188K/E188K*^ hNPCs, 13 DEGs were mis-spliced (*p* = 5.3 × 10^−3^, Fisher’s exact test) compared to 46 mis-spliced DEGs (of 661 DEGs, *p* = 4.2 × 10^−7^, Fisher’s exact test) in *THOC6*^W100*/W100*^ hNPCs, indicating a subtle effect of mis-splicing on expression (Fig. 4A). We found stronger effects on AS and gene expression with the more severe *THOC6* variant, as nearly double the number of AS genes (336 in *THOC6*^*E188K/E188K*^; 661 in *THOC6*^W100*/W100*^) and DEGs (435 in *THOC6*^*E188K/E188K*^; 741 in *THOC6*^W100*/W100*^) were detected in *THOC6* p.W100* nonsense samples relative to p.E188K missense samples (Fig. 4A). Relevant for TIDS pathology, 20% (68 of 336; *p* = 1.5×10^−5^, Fisher’s exact test) of *THOC6*^*E188K/E188K*^ DEGs and 18% (118 of 661; *p* = 9.7×10^−6^, Fisher’s exact test) of *THOC6*^W100*/W100*^ DEGs are syndromic ID genes, which conveys important information pertinent for understanding the pathogenic mechanisms of TIDS (Fig. 4A). DEGs in affected hNPCs are enriched for long genes with an average of less than 10 annotated isoforms (Fig. 4B, C). In addition, 96.45% of downregulated genes in *THOC6* affected hNPCs represent protein-coding genes whereas differentially expressed long non-coding RNAs (lncRNAs) exhibit a trend towards upregulation (comprising 6.29% of the detected upregulated genes) (Fig. 4D). These findings may reflect a requirement for the larger THOC6-dependent TREX tetramer complex function in facilitating mRNP processing of lncRNAs as well as long mRNAs with high expression in the brain.

Intron splicing defects in protein-coding genes (PCGs) generate mRNA that are often unstable^64–67^. In contrast, intron inclusion in lncRNAs can alter nuclear export and structural conformation^64–67^. The THOC6-dependent percentage spliced-in (ΔPSI) was calculated by correlating mRNA transcript fold change with the frequency of intron inclusion. A subtle trend of lower protein coding gene expression was correlated with intron inclusion AS events in *THOC6-*affected genotypes, albeit weakly (slope, *p* = 0.0045 for *THOC6*^W100*/W100*^and *p* = 0.0002 for *THOC6*^*E188K/E188K*^, simple linear regression) (Fig. S4C). Conversely, exclusion of introns in lncRNAs was associated with elevated expression of multiple lncRNAs, including *MEG3* and *MEG8* (Fig. S4C).

To identify functional convergence of *THOC6* DEGs, DAVID analysis was performed to reveal biological categories defined by DEGs in*THOC6* affected hNPCs (Fig. 4E). Downregulated genes are enriched in integrin cell adhesion, extracellular matrix interactions, PI3K-AKT signaling, and TGF-β signaling pathways, which are critical for brain development (Fig. 4E). PI3K-AKT/mTOR signaling regulates cortical NPC proliferation, differentiation, and apoptosis^68,69^. Over 30 genes attributed to the PI3K-AKT/mTOR signaling pathway were downregulated in affected cells, accounting for the significant enrichment in this pathway (*p* = <1 × 10^−13^). *HAPLN1*, *MYC*, *BMPR1B*, *DCN*, *FBN1*, *INHBA*, *ID4*, *THBS1*, *TGFB2*, DEGs enriched in the TGF-β signaling pathway (*p* = <0.001), have direct implications for TGF-β signaling in neural induction, differentiation, and NPC fate specification in TIDS developmental mechanisms^70,71^. Complementary pathways enriched with upregulated DEGs implicate multipotency (*OCT4*, *PAX6*), proliferation, neuron differentiation and WNT signaling pathways (*p* = <1 × 10^−6^, *p* = <0.001, *p* = <0.001, and *p* = <0.001, respectively). WNT signaling is known to promote NPC self-renewal expansion during corticogenesis^72,73^. Shared dysregulation of mTOR, TGF-β, and WNT signaling, coupled with upregulation of multipotency factors in affected genotypes, suggests defects in hNPC multipotency and neural differentiation underlie TIDS pathogenesis.

To refine specific candidate genes implicated in shared TIDS neuropathology, DEGs between affected *THOC6* genotypes were intersected. Twelve genes were upregulated and 117 were downregulated in affected hNPCs, with notable lncRNAs represented. Significant enrichment was detected in Integrin 1 pathway and extracellular matrix protein interaction networks (Fig. S4D). Using mRNA obtained from three independent replicate differentiations of hNPCs per genotype, significant upregulation of *MEG3*, *MEG8*, *ESRG*, and *NEAT1* lncRNAs was confirmed by RT-qPCR (Fig. 4F). RNA FISH confirmed increased expression of *MEG3* in affected hNPCs compared to controls, with elevated signal observed in both nuclear and cytoplasmic compartments (Fig. 4G). Upregulation of functional lncRNAs *NEAT1* and *MEG3* has been linked to the activation of WNT and suppression of TGF-β signaling, respectively^74,75^. Concordant with these findings, the protein level of WNT and TGF-β signaling components in *THOC6-*affected hNPCs exhibit a corresponding differential up- and down-expression relative to controls. Specifically, WNT signaling components WNT7A and TP53 showed increased protein expression, with higher abundance detected in affected hNPCs, together with high expression of OCT4 (Fig. 4H). TGF-β pathway protein HAPLN1 showed reduced protein expression in affected hNPCs, together with lower CEMIP and DKK2 levels (Fig. 4H). We propose that loss of THOC6 leads to lncRNA-mediated dysregulation of key developmental signaling pathways which has implications for the balance of proliferation and differentiation during neural development.

### ***THOC6*forebrain organoid neuropathology impinges on NPCs**

**Fig. 5:**
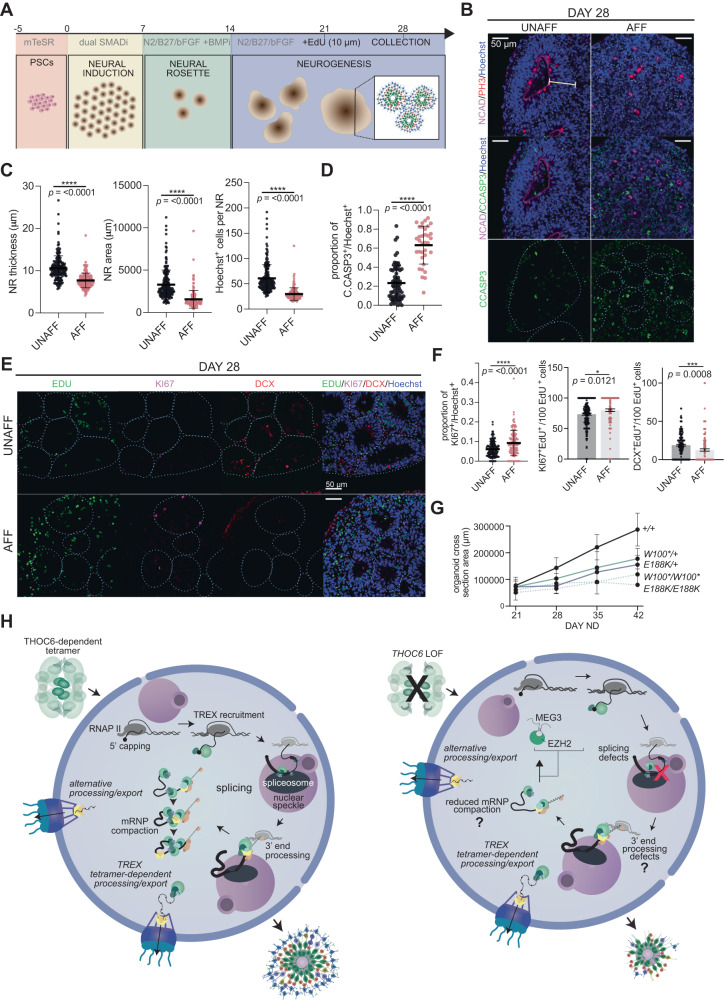
Modeling of *THOC6* variant pathogenesis in human cerebral organoids. **A** Cerebral organoid differentiation protocol. ND, neural differentiation. **B** Immunostaining of PH3, N-Cadherin, apoptosis marker cleaved caspase−3 (C.CASP3), and Hoechst in day 28 human cerebral organoids differentiated from unaffected and affected iPSCs, highlighting differences in neural rosette (NR) morphology. X40 magnification; Scale bar: 50 μm. UNAFF, unaffected; AFF, affected. Representative images for UNAFF and AFF are *THOC6*^*E188K/+*^ and *THOC6*^*E188K/E188K*^, respectively. **C** Quantifications of NR thickness (left), NR area (middle), Hoechst+ cells per NR. UNAFF, unaffected (black; quantifications from *THOC6*^*E188K/E188K*^ and *THOC6*^W100*/W100*^); AFF, affected (red; quantifications from *THOC6*^*E188K/+*^ and *THOC6*^W100*/+^). NR (organoid) biological replicates analyzed across three differentiation replicates per genotype: unaffected, *n* = 67 (15) biological replicates; affected, *n* = 34 (10) biological replicates. Data shown as mean ± SEM. Significance, two-tailed unpaired *t* test; *p* = <0.0001 (thickness), *p* = <0.0001 (area), *p* = <0.0001 (Hoescht + ). **D** Fraction of C.CASP3+ cells per NR for *THOC6*^W100*/+^ and *THOC6*^*E188K/+*^ controls and *THOC6*^W100*/W100*^ and *THOC6*^*E188K/E188K*^ affected organoids. UNAFF, unaffected (black; quantifications from *THOC6*^*E188K/E188K*^ and *THOC6*^W100*/W100*^); AFF, affected (red; quantifications from *THOC6*^*E188K/+*^ and *THOC6*^W100*/+^). NR (organoid) biological replicates analyzed across three differentiation replicates per genotype: unaffected, *n* = 67 (15) biological replicates; affected, *n* = 34 (10) biological replicates^.^ Data shown as mean ± SEM. Significance, two-tailed unpaired *t* test, *p* = <0.0001. **E** Immunostaining of EDU, KI67, DCX to assess timing of differentiation in day 28 organoids. Representative images for UNAFF and AFF are *THOC6*^*E188K/+*^ and *THOC6*^*E188K/E188K*^, respectively. **F** Quantifications for **E**; UNAFF, unaffected (black; quantifications from *THOC6*^*E188K/E188K*^ and *THOC6*^W100*/W100*^); AFF, affected (red; quantifications from *THOC6*^*E188K/+*^ and *THOC6*^W100*/+^). NR (organoid) biological replicates analyzed across three differentiation replicates per genotype: unaffected, *n* = 187 (87) biological replicates; affected, *n* = 157 (67) biological replicates. Data show*n* as mean ± SEM. Significance, two-tailed unpaired *t* test; *p* = <0.0001 (KI67 + /Hoescht + ), *p* = 0.0121 (KI67+EdU + / EdU + ), *p* = 0.0008 (DCX+EdU + /EdU + ). **G** Growth rate of organoids across genotypes measured by cross section area (μm) from days 21-42. Genotypes: *THOC6*^*+/+*^ (black), *THOC6*^*E188K/+*^ (dark purple), *THOC6*^*E188K/E188K*^ (purple), *THOC6*^W100*/+^ (dark green), *THOC6*^W100*/W100*^ (green). **H** Schematic of proposed model of *THOC6* pathogenesis, depicting disruption of RNA processing in human neural cells leading to impaired proliferation and differentiation timing during corticogenesis. Additionally, given ALYREF’s defined role in multimerizing several TREX complexes coating mRNPs^33^, impaired recruitment of ALYREF to TREX complexes has implications for mRNP compaction in THOC6 LOF cells.

*THOC6* pathogenesis in human cortical development was investigated using dorsal forebrain-fated organoids neurally differentiated from iPSC lines (Fig. 5A). Forebrain organoids recapitulate the cellular heterogeneity and developmental dynamics of early corticogenesis^76^. Within each organoid, several neural rosette (NR) structures develop stochastically to recapitulate features of in vivo ventricular zone development, including hNPC proliferation and differentiation to cortical neuron fates (Fig. 5A). NR morphology was evaluated in cortical organoids at 28 days in neural differentiation (ND) from three independent differentiations per genotype. To minimize the effect of genetic background-dependent NR variability, the following analyzes focus on heterozygous unaffected and homozygous affected familial comparisons. To characterize the NR proliferative niche, the maximum thickness of the NR neuroepithelial center was measured as defined by N-cadherin immunostaining and pseudostratified NR cytoarchitecture by Hoechst staining (Figs. 5B, C, S5A, and B). *THOC6*-affected organoids show significantly thinner pseudostratified neuroepithelium (*p* = <0.0001, two-tailed *t* test), concordant with reduced NR size composed of fewer cells (*p* = <0.0001, two-tailed *t* test) (Figs. 5B, C, S5A, and B). In addition, we observed a significantly higher proportion of affected NR cells expressing the apoptotic marker cleaved caspase-3 (C.CASP3) compared to unaffected NR cells (*p* = <0.0001, two-tailed *t* test) (Fig. 5D), evidence that supports apoptosis as a mechanism of reduced NR size in the *THOC6*-affected organoids (Figs. 5B, D, S5A, and B).

To assess alterations in the timing of differentiation in affected NRs, we performed EdU-pulse labeling at day 21 ND for 24 hours to label mitotically active cells, followed by organoid immunohistochemistry analysis at day 28 ND (Figs. 5E, F, S5C, and D). To assess the balance of multipotency and differentiation, cells co-labeled with the proliferation markers EdU and KI67, and the migrating neuron marker doublecortin (DCX) were quantified per NR. A significant increase in cells co-stained with KI67 and EdU per affected NR was detected at day 28 ND (*p* = <0.0001, two-tailed *t* test), indicating affected NPCs remain mitotically active longer than control NPCs (Fig. 5F). This finding, paired with the elevated mRNA and protein expression of OCT4 in affected hNPCs (Fig. 4H), supports a retention of multipotency model. Consistent with this finding, we observed a significant reduction in the fraction of EdU cells co-labeled with DCX in affected NRs (*p* = <0.0001, two-tailed t-test) (Fig. 5F). Together with the prolonged proliferation dynamics, this finding suggests a disruption to the differentiation timeline in affected organoids.

To investigate the effects of reduced NR growth on organoid size, we measured whole organoid cross-section areas weekly from day 21 to day 42. Compared to the steady size increase of *THOC6*^*+/+*^ organoids, affected organoids showed a slower growth rate (*THOC6*^*E188K/E188K*^: *p* = 0.0122; *THOC6*^W100*/W100*^: *p* = 0.0362) (Fig. 5G). Together, our findings implicate a pathogenic mechanism of delayed differentiation due to reduced NPC proliferative capacity and elevated apoptosis with subsequent cortical growth impairment in affected organoids.

### ***Thoc6*is required for mouse embryogenesis**

**Fig. 6:**
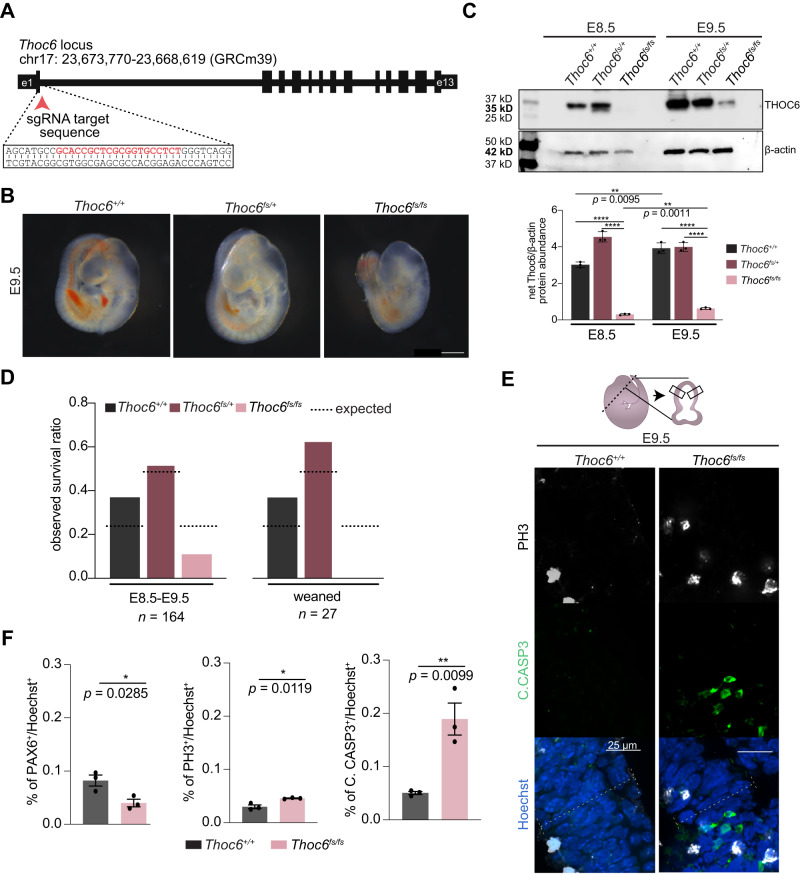
Generation of *Thoc6*^*fs/fs*^ mouse model. **A** CRISPR/Cas9 editing strategy to introduce frameshift variants in mouse *Thoc6*. sgRNA sequence highlighted in red text. **B** Representative images of isolated *Thoc6*^*+/+*^, *Thoc6*^*fs/+*^, and *Thoc6*^*fs/fs*^ whole embryos at E9.5. Scale bar: 50 μm. **C** Western blot analysis with quantifications of THOC6 levels in E8.5 and E9.5 mouse embryos. β-actin, loading control. Genotypes: *Thoc6*^*+/+*^ (black), *Thoc6*^*fs/+*^ (dark red), *Thoc6*^*fs/fs*^ (pink). Data shown as mean ± SEM. Significance, two-tailed *t* test, **** indicates *p* = <0.0001. This experiment was repeated three times independently and showed similar results. **D** Litter ratio analysis for E8.5–9.5 (left) and weaned (right) *Thoc6*^*fs/fs*^ mice. Ratios are consistent with embryonic lethality of homozygous frameshift mice. Genotypes: *Thoc6*^*+/+*^ (black) *Thoc6*^*fs/+*^ (dark red), *Thoc6*^*fs/fs*^ (pink). *N* = 164 mice (left); *n* = 27 mice (right). **E** Immunostaining of markers PH3 and C.CASP3 in E9.5 mouse forebrain. Illustration highlights sectioning and quantification approach. 60x magnification; Scale bar: 25 μm. **F** Quantifications of fractions of PAX6, PH3, and C.CASP3-expressing cells in E9.5 neuroepithelium. Measurements were combined from one rostral and one caudal section (from two lateral segments depicted by solid black boxes in E). *N* = 3 embryo replicates per genotype. Genotypes: *Thoc6*^*+/+*^ (black) and *Thoc6*^*fs/fs*^ (pink). Data shown as mean ± SEM. Significance, two-tailed *t* test. Source data are provided as a Source Data file.

To investigate the role of Thoc6 in an in vivo mammalian model, we created a *Thoc6* mouse model. An insertion was introduced into exon 1 of *Thoc6* using CRISPR/Cas9 genome editing. The frameshift variant generates a premature stop codon after 8 amino acids (NM_001008425:r.16_39del;r.39_40ins[40-2582_40-2562], p.P6Lfs*18, referred to as *Thoc6*^*fs*^) (Figs. 6A and S6A, B). *Thoc6*^*+/fs*^ do not exhibit overt phenotypes compared to *Thoc6*^*+/+*^ littermates, but heterozygous crosses do not yield homozygous offspring at birth. Analysis of *Thoc6*^*+/fs*^ time pregnant litters confirmed that *Thoc6*^*fs/fs*^ pups are embryonic lethal. Curvature of the embryonic trunk and axial rotation persists in *Thoc6*^*fs/fs*^ littermates, consistent with normal differentiation of germinal layers during development. Phenotypic differences between wildtype (WT) and *Thoc6*^*fs/fs*^ embryos were noted starting at E7.5. By E9.5, *Thoc6*^*fs/fs*^ embryos were smaller with delayed development. Genotypic dependent defects in neocortical development were particularly pronounced (Fig. 6B). At E8.5, THOC6 was undetectable in *Thoc6*^*fs/fs*^ mouse embryos relative to control littermates. Of note, there is a faint minor allele product visible in *Thoc6*^*fs/+*^, likely representing a product ~24 amino acids shorter generated from an alternative translation initiation site 73 nucleotides downstream of the wildtype start site (Fig. 6C). By E9.5, a developmental time point with high THOC6 expression, the minor allele product is faintly detectable in *Thoc6*^*fs/fs*^ embryos by Western blot (Fig. 6C). Embryonic lethality was confirmed by E11.5, indicating one functional allele of *Thoc6* is essential for mouse embryonic development (Fig. 6C, D).

Forebrain tissues of *Thoc6* E8.5-E10.5 littermates revealed consistently thinner neuroepithelium (PAX6^+^) in *Thoc6*^*fs/fs*^ telencephalic vesicles compared to *Thoc6*^*+/+*^ littermate controls (Figs. 6E, 6F, and S6C–E). Additionally, E9.5 *Thoc6*^*fs/fs*^ neuroepithelium had an increase in mitotically active cells (PH3^+^) compared to *Thoc6*^*+/+*^ littermates, as well as widespread apoptosis (C.CASP3^+^), providing evidence for a shared mechanism of altered corticogenesis in mammals (Fig. 6E, F). These findings are consistent with a requirement for Thoc6 in expansion of the neural epithelium, indicating that THOC6 is important for mammalian corticogenesis.

### THOC6 molecular functions are conserved in mouse

**Fig. 7:**
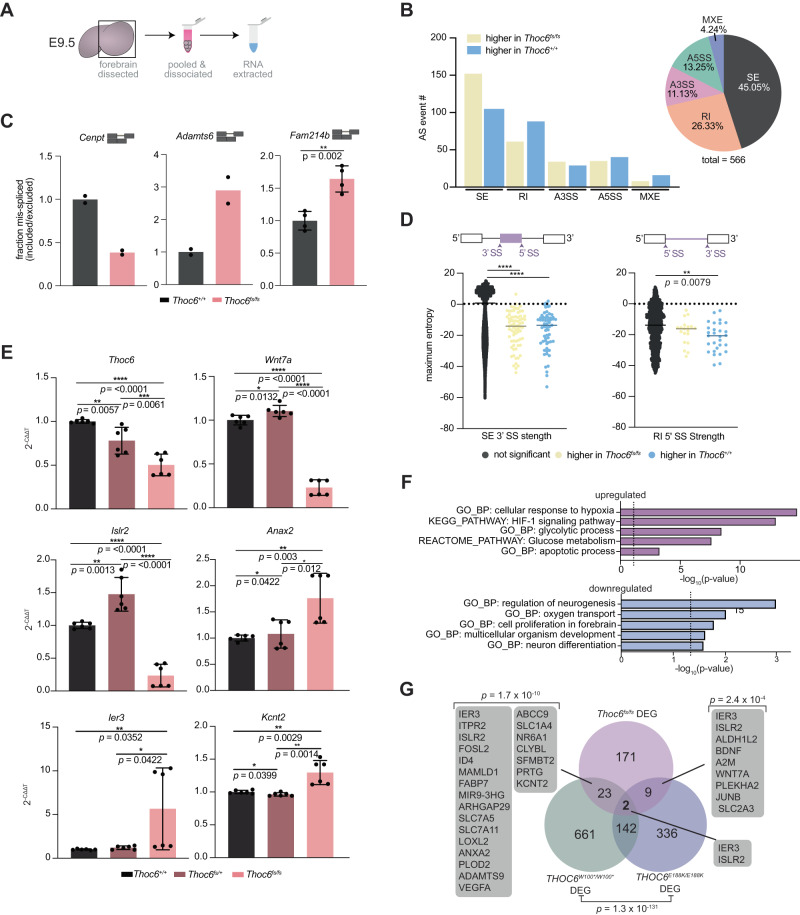
Characterization of mRNA processing defects in *Thoc6*^*fs/fs*^ mouse E9.5 forebrain. **A** Cartoon of E9.5 mouse forebrain total RNA sample preparation. **B** rMATS summary results of statistically significant AS events in *Thoc6*^*fs/fs*^ E9.5 forebrain. Event type (pie chart) and inclusion status (bar chart). Yellow, higher inclusion in *Thoc6*^*fs/fs*^. Blue, higher inclusion in *Thoc6*^*+/+*^. **C** Quantifications from RT-PCR validating top AS events *Cenpt* and *Admts6* (*n* = 2 biological replicates per genotype), and *Fam214b* (*n* = 4 biological replicates per genotype). Genotypes: *Thoc6*^*+/+*^ (black) and *Thoc6*^*fs/fs*^ (pink). Data are mean ± SEM. Significance for *Fam214b* determined by two-tailed unpaired *t* test, *p* = 0.002. **D** Significant splice site strength score differences at mis-spliced events in *Thoc6*^*fs/fs*^ samples based on maximum entropy model. Black, non-significant events. Yellow, higher inclusion in *Thoc6*^*fs/fs*^. Blue, higher inclusion in *Thoc6*^*+/+*^. Data are mean ± SEM. Significance, two-tailed unpaired *t* test. SE 3’ SS strength (**** indicates *p* = <0.0001). RI 5’ SS strength (*p* = 0.0079). **E** RT-qPCR validations of *Thoc6*, *Wnt7a*, *Islr2*, *Ier3*, *Kcnt2*, *Anax2* mRNA abundance on two additional biological E9.5 forebrain replicates per genotype. Genotypes: *Thoc6*^*+/+*^ (black) and *Thoc6*^*fs/fs*^ (pink). *N* = 3 experimental replicates per forebrain specimen (total of *n* = 6 per ge*n*otype). Data are mean ± SEM. Significance, two-tailed unpaired *t* test. *P*-values for in order of comparison (*Thoc6*^*+/+*^ vs. *Thoc6*^*fs/fs*^, *Thoc6*^*fs/+*^ vs. *Thoc6*^*fs/fs*^, *Thoc6*^*+/+*^ vs. *Thoc6*^*fs/+*^), per gene: *Thoc6* (*p* = <0.001, 0.0061, 0.0057), *Wnt7a* (*p* = <0.001, <0.0001, 0.0132), *Islr2* (*p* = <0.001, <0.0001, 0.0013), *Anax2* (*p* = 0.003, 0.012, 0.0422), *ler3* (*p* = 0.0352, *p* = 0.0422), *Kcnt2* (*p* = <0.0029, 0.0014, 0.0399). This experiment was repeated twice independently and showed similar results. **F** DAVID analysis shows significantly enriched biological pathways among upregulated genes (top, magenta) and downregulated genes (bottom, blue) in *Thoc6*^*fs/fs*^ E9.5 forebrain. Significance, one-sided Fisher’s Exact test. **G** Venn diagram of the overlap DEG in affected hNPCs and *Thoc6*^*fs/fs*^ mouse E9.5 forebrain. Overlap significance tested by one-sided Fisher’s exact test.

To investigate THOC6-dependent TREX functions that account for phenotypic differences between humans and mice with THOC6 LOF variants, mRNP processing of *Thoc6*^*fs/fs*^ E9.5 mouse neuroepithelium was assessed (Figs. 7A, S6F, G, and S7). Three biological replicates were analyzed by RNAseq per genotype (*Thoc6*^*+/+*^, *Thoc6*^*fs/+*^, *Thoc6*^*fs/fs*^) (Fig. S7A). Fewer significant AS events (FDR < 0.05) were detected in *Thoc6*^*fs/fs*^ samples, as compared to *THOC6* affected hNPCs. Yet, the kind of AS events were consistent between species, with SE (45%) and RI (26%) being the most frequently detected (Fig. 7B and Supplementary Data 3). Events with greater than 40 PSI were quantified in the *Thoc6*^*fs/fs*^ transcriptome, and RI AS events in *Cenpt*, *Adamts6*, and *Fam214b* were validated (Figs. 7C and S7B). Applying the maximum entropy model analysis of splice junctions revealed significantly weaker 3’ splice site strengths for SE events and weaker 5’ splice sites associated with RI events in the *Thoc6*^*fs/fs*^ mouse model. The number of AS events and signature of splice site weakness was more modest in the mouse model compared to hNPC models, which might reflect differences in NPC purity between forebrain tissues and stem cell-derived hNPCs. However, collectively, this shared mis-splicing signature suggests a conserved role of THOC6-dependent TREX tetramer in coordinating mRNA processing that precedes TREX export functions (Figs. 7D and S7C).

Notably, biological pathway and network enrichment analysis of AS genes identified mRNA processing, pre-miRNA processing, de-adenylation of mRNA, central nervous system development, forebrain development, multicellular growth, response to oxidative stress, cytoskeletal organization, and neuron projection (Fig.p S7D) — several of the biological categories associated with hNPCs ASGs. These shared findings suggest selective conservation of mRNP processing mechanisms by *THOC6* in mouse and human forebrain.

To assess the correlation between THOC6 mRNP processing defects and gene expression, differential expression analysis of *Thoc6*^*fs/fs*^ forebrain RNAseq data was performed compared to *Thoc6*^*+/+*^ controls. *Thoc6* mRNA was downregulated two-fold in *Thoc6*^*fs/fs*^ mutant mouse forebrain compared to control (*p* = <0.0001) (Figs. 7E and S7F). In the *Thoc6* mouse model, 5x more genes were upregulated (144 genes) than downregulated (27 genes). Nevertheless, downregulated genes may convey important pathology. First, downregulated genes functionally converge on neurogenesis, proliferation, and differentiation pathways (Fig. 7F). Upregulated genes are implicated in the hypoxic response, HIF-1 signaling pathway, and glycolysis—biological categories indicative of increased apoptosis in affected cells (Fig. 7F). These results are consistent with the observation of apoptosis in *Thoc6*^*fs/fs*^ E9.5 neuroepithelium (Fig. 6E, F).

DEGs shared between mouse and human model systems are consistent with conserved TIDS molecular pathology (Fig. 7G). More *Thoc6*^*fs/fs*^ DEGs overlapped with *THOC6*^W100*/W100*^ (23 genes) than *THOC6*^*E188K/E188K*^ (9 genes) samples, and include genes involved in neurogenesis, hypoxic response, and synapse regulation. Validation of *Ier3*, *Islr2, Wnt7a*, *Kcnt2*, *Anax2*, and *Vegfa* DEGs shared across affected models were confirmed by RT-qPCR in three additional E9.5 forebrain biological replicates for *Thoc6*^*+/+*^, *Thoc6*^*fs/+*^, and *Thoc6*^*fs/fs*^ samples (Figs. 7E and S7G). Overlapping affected human and mouse molecular mechanisms suggest shared pathology. However, the extent of upregulation of genes in response to increased apoptosis is exacerbated in mouse, highlighting species-specific phenotypic differences due to loss of THOC6.

## Discussion

A growing cohort of individuals with TIDS are being identified by exome-based genetic testing^6,9–19,48,77^, highlighting important molecular functions of THOC6 and the TREX complex^25,34,36,52–54,78–88^. Our findings revealed a novel *THOC6* LOF model of TIDS, characterized by stable mRNA transcribed from pathogenic *THOC6* alleles that is translated to missense (p.E188K) and a low abundance product (p.W100*). Our findings suggest that variant THOC6 proteins do not facilitate THO/TREX tetramer complex formation. While mRNA export was unchanged in *THOC6-affected* NPCs, mis-splicing was observed, implicating nonredundant functions between TREX dimer and tetramer complexes. Alternative splicing preferentially disrupts the processing of long mRNAs and long genes expressed in the brain. Alternative splicing defects were conserved in the *Thoc6* LOF mouse model. Dissimilar to TIDS, *Thoc6*^*fs/fs*^ pups were embryonic lethal by E10.5, revealing organism-specific tolerance to loss of THOC6.

The first THOC6 human genetic study identified a founder *THOC6* triple-variant haplotype (TVH), *THOC6* c.[298 T > A;700 G > C;824 G > A], (p.[W100R;V234L;G275D]) that segregates in individuals of European ancestry^10,14,18,77^. We identified siblings of South Asian ancestry with classic TIDS that are biallelic for one of the TVH variants (*THOC6* c.824 G > A, p.G275D), with confirmed absence of the other two TVH variants (*THOC6* genotypes: c.[298 T/T; 700 G/G]). Comparing these two haplotypes provides evidence for the pathogenicity of the *THOC6* p.G275D variant but does not negate the predicted pathogenicity of the corresponding p.W100R or p.V234L TVH variants. Comparable clinical phenotypes between haplotypes with one versus three missense variants suggest a single THOC6 variant is sufficient to comprehensively disrupt THOC6, a baseline deficiency not exacerbated by the accumulation of additional LOF variants.

The shared clinical phenotypes between biallelic *THOC6* nonsense and missense variants indicates that *THOC6* variants generally function through a LOF genetic mechanism, with both low protein abundance and normal expression of variant THOC6 disrupting TREX tetramer complex formation. Given the conserved molecular functions of TREX in mammals, the phenotypic discrepancy between *Thoc6*^*fs/fs*^ mouse embryonic lethality and human biallelic *THOC6* TIDS features is notable, suggesting species-specific aspects of THOC6 pathogenic mechanisms. Superficially, this finding suggests that humans are more tolerant of *THOC6* variants. Alternatively, this phenotypic discrepancy may reflect the placement of the frameshift variant in exon 1 of *Thoc6* in the mouse model. Human pathogenic *THOC6* variants reside in the WD40 repeat domains downstream of exon 1. If and how variants impact mRNA stability is not clear, but *THOC6* affected transcripts were not differentially expressed, as determined by both RT-qPCR and RNAseq in independent replicates of hNPCs and iPSCs, while *Thoc6*^*fs*^ transcripts were found to be decreased. It will be important to deconvolve these confounding factors to identify species-specific sensitivity to biallelic *THOC6* pathogenic alleles.

The crystal structure of human TREX demonstrates that THOC6 is required for tetramer formation (Fig. 5H)^25,33^. Defects in TREX tetramer assembly are not predicted to disrupt the formation of stable dimers, allowing THOC6-depleted models to discriminate between dimer and tetramer functions. It is conceivable that TREX dimers retain mRNP functions in metazoans, whereas THOC6-dependent TREX tetramers enhance the efficiency and coordination of these activities. TREX has a prominent role in nuclear RNA export^52,83^, however, the absence of global mRNA export defects in THOC6 LOF hNPCs suggests that THO dimers in THOC6 LOF hNPCs maintain their conserved function for RNA export. The significant splicing changes implicate a pathogenic mechanism whereby THOC6-dependent disruption of TREX tetramer formation indirectly disrupts coordination of co-transcriptional mRNA and lncRNA processing, upstream of poly **A** + RNA packaging and export. This interpretation is supported by the diversity of RNA processing functions attributed to TREX tetramer-associated cofactors, such as UAP56 and ALYREF that play important roles in mediating pre-mRNA splicing decisions^52,89^. Our results do not rule out the possibility that THOC6 plays a direct role in mRNA splicing, outside of mediating TREX core tetrameric assembly. THO member THOC5 has been shown to interact with unspliced transcripts^36^, and WD40-repeat domains facilitate splicing factor interactions with pre-mRNA^56^, which are two lines of evidence in support of this possibility. Additionally, our finding of reduced recruitment of ALYREF to TREX complexes in THOC6 LOF models also has implications for mRNA compaction and organization in affected cells, as evidenced by recent in vitro work showing that globular compaction of human mRNPs occurs via several TREX complexes coating a single mRNP with EJCs multimerizing through ALYREF^33^ (Fig. 5H).

Evolution of the TREX tetramer overlaps with enhanced splicing requirements due to the phylogenetic (yeast versus mammals) increase in the expression of long genes with multiple isoforms, which is particularly relevant in the mammalian brain. Splicing cofactors are known to compete for the limited number of UAP56 binding sites^34,90–92^. The tetramer organization offers additional competitive binding sites to splicing cofactors, promoting coordination of splicing of long transcripts. The tetramer also affords a greater surface area to maintain the structural integrity of long mRNA transcripts to prevent the formation of DNA-RNA hybrid or R-loop structures^39,40^. An indirect scaffolding function is supported by enrichment of aberrant splicing events at weaker splice sites. Weak splice sites are most often utilized by transcripts during alternative splicing, and genes with elevated isoform diversity from alternative splicing are more susceptible to disruption of the overall integrity of RNA processing in *THOC6*-affected hNPCs. Lastly, previous findings indicate that RI events account for a substantial portion of splicing variation in the primate prefrontal cortex, a trend that is most pronounced in humans^93^. Although intron retention is a known mechanism of mouse neuronal gene regulation by initiating RNA exosome-mediated degradation^94^, it is possible that human cells are more tolerant than mouse cells to elevated intron retention. Further investigation of these interspecific differences is important for generating translationally relevant discoveries.

The number of ID genes that are mis-spliced in *THOC6* affected hNPCs relative to controls implicate shared underlying developmental mechanisms of ID pathology. However, the developmental impact of individual processing defects on TIDS neuropathology is complicated by the compounding effects of constitutive THOC6 LOF models. In addition to trends shared with the mouse model, we show that biallelic THOC6 LOF is responsible for the disruption of key TGF-β and Wnt signaling pathways via a mechanism that involves dysregulation of signaling components, and lncRNAs resulting in delayed hNPC differentiation, prolonged retention of multipotency, and enhanced apoptosis. This is exemplified by intron retention and upregulation of *MEG3* in affected hNPCs. *MEG3* is linked to the regulation of TGF-β signaling and other EZH2 common target genes^75^. Our findings suggest that RI events alter *MEG3* subcellular localization, expression, and downstream WNT signaling that increases multipotency and disrupts the balance of proliferation and differentiation in affected hNPCs. A shift towards cytoplasmic localization of lncRNAs has evolved in human cells, which is important for the maintenance of stem cell pluripotency (e.g., cytoplasmic *FAST* binds E3 ubiquitin ligase β-TrCP to block its interaction with β-catenin and enable activation of Wnt signaling)^95,96^. Given the increased diversity of lncRNA functions in human developmental biology, mouse cells may be less tolerant to lncRNA dysregulation than human cells. In addition, *MEG3* is also upregulated by CREB^97^ whose target genes are affected in *Thoc5* conditional knockout mouse cortical neurons^88^, potentially reflecting a shared mechanism of THO dysregulation in neural cells. While our analyzes from mouse and human organoid models of *Thoc6* and *THOC6* disruption provide insight into the molecular pathology of early neural development, later analysis of synaptic physiology will be important to elucidate mechanisms of neuronal dysfunction in TIDS.

## Methods

### Human participants

All participants or parents/guardians in this study were consented under an approved institutional review board. In all cases, the procedures followed were in accordance with the ethical standards of the respective institution’s committee on human research (Radboud University Medical Centre Nijmegen, The Netherlands (Family **F** 1); Marmara University Hospital Pediatric Allergy and Immunology, Istanbul, Turkey (F2); Greenwood Genetic Center, Greenwood, South Carolina, USA (F3); Kasturba Medical College, Manipal, India (F4); Imagine Institute, Paris, France (F5); Case Western Reserve University, OH, USA (F6-F7)) and were in keeping with international standards. Probands (P) 1-3 and 5 were identified through GeneMatcher and personal communications^98^. The assigned sex at birth for Probands 1, 3, 6, and 7 was male. The assigned sex at birth for Probands 2, 4.1, 4.2, and 5 was female. Consent for publishing individual-level data was provided by all participants and/or parents/guardians. The authors affirm that all human research participants [or their parents/guardians] provided informed consent for the publication of the images in Fig. 1. Sex-influenced phenotypes relevant to the TIDS clinical report include genitourinary defects. No further sex- or gender-based analysis were performed given that this manuscript is focused on neurodevelopmental defects which are not influenced by sex/gender. Details for all individuals are provided in Table S1.

### Animal models

All mice were maintained according with the National Institutes of Health Guidelines for the Care and Use of Laboratory Animals and were approved by the Case Western Reserve Institutional Animal Care and Use Committee. CRISPR genome editing was performed in the University of California, San Diego Transgenic and Knockout Mouse Core. C57BL/6JN hybrid mice (Jackson Laboratory, 005304) were used for CRISPR editing of the *Thoc6* locus. Founder mice with the *Thoc6*^*fs/+*^ allele were intercrossed with C57BL/6JN mice (Jackson Laboratory, 005304) for line maintenance. All ex vivo analyzes were performed on tissue collected from mice of both sexes at embryonic day **E** 8.5-10.5. Sex-dependent differences were not assessed due to the focus of this manuscript is on neurodevelopmental defects in TIDS, which are not influenced by sex/gender.

Litters were genotyped by allele-specific polymerase chain reaction (AS-PCR). Genomic DNA was prepared from mouse tissue samples as previously described^99^. AS-PCR for each allele was assembled using the standard GoTaq DNA polymerase (Promega) protocol. Reaction conditions were executed as recommended by the manufacturer. Primers and sgRNA sequences are provided in Table S2.

### Human ESC/iPSC culture

Human ESC and iPSC lines were cultured using feeder-free conditions on Matrigel (Corning) with mTeSR-1 (STEMCELL Technologies). Commercial lines used in this manuscript include: H9 (THOC6 + / + , 46XX, ESCs, WA09, WiCell). The following lines used in this manuscript were established as part of the Diabetes iPSC Panel by the New York Stem Cell Foundation (NYSCF) and is available to researchers through the biorepository at NYSCF (nyscf.org/research-institute/repository-stem-cell-search/): AS0035 (THOC6 + / + , 46XX, iPSCs, NYSCF Diabetes iPSC Panel), and AS0041 (THOC6 + / + , 46XY, iPSCs, NYSCF Diabetes iPSC Panel). The following iPSC lines were reprogrammed from primary skin fibroblasts: [KMC6002 (*THOC6*^*E188K/+*^, 46XY, iPSCs), KMC6003 (*THOC6*^*E188K/E188K*^, 46XY, iPSCs), KMC7001 (*THOC6*^W100*/+^, 46XY, iPSCs), KMC7002 (*THOC6*^W100*/W100*^, 46XY, iPSCs)]. Lines KMC6002 and KMC6003 were reprogrammed from primary skin fibroblasts obtained from individual P6 and the unaffected father. Lines KMC7001 and KMC7002 were reprogrammed from primary skin fibroblasts obtained from individual P7 and the unaffected father. Mycoplasma-free fibroblast lines were episomally reprogramed. The lines were balanced for sex and affectation status, consisting of a control male and female and an affected male and female. iPSC lines were established from reprogramming of 1×10^6^ fibroblasts. Lines were expanded and characterized from each genotype. The pluripotent and genomic integrity of these lines were characterized for: (1) Morphology and growth rate: iPSCs exhibit a characteristic cell morphology of tightly packed colonies with sharply demarcated borders with a doubling rate of approximately 24 hours. Cell lines that do not meet these criteria are not analyzed. (2) Karyotype: iPSCs frequently acquire extra chromosomes, especially of chromosomes 12, 17, and X. Metaphase spreads of chromosomes were performed by Cell Line Genetics to ensure euploidy. (3) Expression of pluripotency markers: We confirmed the presence of the cell surface pluripotency markers by IHC. Expression of NANOG, TRA-1-81, LIN28 and TRA-1-60 was confirmed in each iPSC line with commercially available antibodies. Cells are not used past passage 45.

Passaging was performed using mTeSR-1 supplemented 1 nM ROCK inhibitor (BD Biosciences 562822) to prevent differentiation. Both manual and chemical dissociation with Versene (Gibco 15040066) were performed for splitting. Sanger sequencing validation of genotypes from DNA (Fig. S1A and B) and cDNA (Fig. S1D) (primers listed in Table S2), as well as CNV microarray analysis (Illumina Bead Array, analysis with Genome Studio v2.0), were routinely performed on all lines to ensure no pathogenic changes or cellular contamination occurred during culturing.

### Whole exome sequencing and analysis

Exome libraries from the genomic DNA of all participants were prepared and captured with the Agilent SureSelectXT Human All Exon 50 Mb Kit for individuals P1 & P4-P7, the Agilent SureSelectXT Clinical Research Exome kit for P3, and the TrueSeq Rapid Exome Kit for P2. Further, exome libraries were sequenced on an Illumina HiSeq or NextSeq instrument^100^.

Reads were aligned to the human reference genome NCBI builds 37 (GRCh37) 38 (GRCh38) and 38 using Burrows-Wheeler Aligner (BWA)^101^. Variant calling of single nucleotide variants (SNVs) and copy number variants (CNVs) was performed using GATKv4^102^, VEP, and CoNIFERv0.2.2^103^. The average depth of coverage was calculated across all targeted regions. The data were filtered and annotated relative to the canonical *THOC6* transcript (ENST00000326266.8) and protein (ENSP00000326531.8) using in-house bioinformatics software^104^. Variants were also filtered against public databases including the 1000 Genomes Project phase 311, Genome Aggregate Database (gnomADv3), National Heart, Lung, and Blood Institute Exome Sequencing Project Exome Variant Server (ESP6500SI-V2). Those with a minor allele frequency >3% were excluded. Additionally, variants flagged as low quality (Phred quality score <30) were excluded from the analysis. Variants in genes known to be associated with ID were selected and prioritized based on predicted pathogenicity.

### Sanger sequencing

All variants discovered by WES that mapped to *THOC6* (NM_024339.5) were confirmed with Sanger sequencing for each individual and respective family members who submitted samples, except for P1 where high-coverage WES of *THOC6* in the proband and parents was deemed sufficient to report without Sanger confirmation. Chromatograms were analyzed using Sequencer (v5.4.6) and Geneious Prime Software (v.2022.1.1).

### Cerebral organoid generation

Telencephalic cerebral organoids were generated based on previously published protocols^105^, with few modifications to start with low cell density in order to generate smaller and more consistent embryoid bodies (EBs). Briefly, human ESCs/iPSCs were passaged into 96-well V-shaped bottom ultra-low attachment cell culture plates (PrimeSurface® 3D culture, MS-9096VZ) to achieve a starting cell density of 600–1000 cells per well in 30 µl of mTesR^TM^1 with 1 nM ROCK inhibitor. After 36 hours, 150 µl of N-2/SMAD inhibition media (cocktail of 1X N-2 supplement (Invitrogen 17502048), 2 μM A-83-01 inhibitor (Tocris Bioscience 2939), and 1 mM dorsomorphin (Tocris Bioscience 309350) in DMEM-F12 (Gibco 11330032)) was added for neural induction. On day 7, EBs were transferred to Matrigel-coated plates to enrich for neural rosettes at a density of 20-30 EBs per well of a 6-well plate, and media was changed to neural differentiation media (0.5X N-2 supplement, 0.5X B-27 supplement (Invitrogen 17504044) with 20 pg/μl bFGF and 1 mM dorsomorphin inhibitor in DMEM/F-12). For organoid differentiation EBs were outlined on day 14 using a pipet tip and uplifted carefully with a cell scraper to minimize organoid fusion and tissue ripping. Media was changed once more to N-2/B-27 with bFGF only and plates with uplifted organoids were placed on a shaker in the incubator set at a rotation speed of 90. On day 14, media was changed once more to N-2/B-27 with bFGF only. Prior to day 14, media changes were performed every 48 hours. After day 14, daily media changes were performed until collection. For monolayer NPC differentiation, neural rosettes were scored and uplifted on day 14, dissociated in Accutase (Gibco A1110501), and re-plated on poly-L-ornithine (PLO)/Laminin-coated plates for NPC expansion, selection, and passaging. 15 μg/mL PLO (Sigma-Aldrich P4957) diluted in DPBS (Gibco 14040-133); 10 μg/mL laminin (Sigma-Aldrich L2020) diluted in DMEM/F-12.

### HEK 293T transfection and overexpression assay

HEK 293 T cells were plated in DMEM + 10% fetal bovine serum (FBS) at a density of 1 × 10^5^ cells per dish of a 12-well tissue culture plate. After 24 hours, 2 µg of the pcDNA™5/FRT/TO-Thoc6-Flag expression vector and 8 µl polyethylenimine (PEI) were mixed in 100 µl OPI-MEM media and added to cell culture media. After 24 hours post transfection, media was changed to DMEM + 10% FBS medium with 200 ng/mL doxycycline to induce Thoc6-Flag expression. At 48 hours post transfection, cells were collected for western blot analysis (Fig. S2A).

### Western blot analysis and immunoprecipitation

ESCs, iPSCs, and NPCs used for western blot analysis were pelleted and lysed in RIPA buffer supplemented with 1:50 protease inhibitor cocktail (Sigma-Aldrich P8340) and 1:100 phosphatase inhibitor cocktail 3 (Sigma-Aldrich P0044) using mortar and pestle coupled with end-over-end rotation for 30 minutes to 1 hr at 4 °C, or sonication. Protein concentration was quantified by BCA (Thermo Scientific Pierce A53227). Lysis samples were then incubated at a 1:3 ratio with 4x Laemmli sample buffer (Bio-Rad) supplemented with 10% BME and incubated at 95-110 °C on a heat block for 5 minutes for denaturation. For co-immunoprecipitation, primary antibodies anti-THOC5 and anti-THOC6 (1:50 dilution in 1x PBS with Tween-20) were incubated overnight at 4 °C with Dynabeads Protein G (Invitrogen, 10003D). Beads were washed and cell lysis (35 μg of protein) was added for incubation overnight at 4 °C with rotation. IP samples were prepared according to the manufacturer’s instructions with elution in 4x Laemmli sample buffer with 10% BME. For promotion of readthrough of premature termination codons, Ataluren (eMolecules NC1485023) was dissolved in DMSO added to ESC/iPSC media at a final concentration of 30 μM for 48 hours^106^. Protein was then extracted as described above.

Samples were loaded into 4-20% SDS-polyacrylamide precast gels (Bio-Rad) and proteins were separated by electrophoresis at 30 V for ~4 hours room temperature. Separated proteins were then transferred to PVDF membranes (Millipore) overnight using a wet transfer system (Bio-Rad) at 4 °C, or the Bio-Rad Trans-Blot Turbo transfer system. For immunoblotting, membranes were incubated in 5% milk-blocking buffer (1x TBS-T) followed by primary antibody incubation overnight at 4 °C with rotation. Membranes were washed 3 times for 5 minutes in 1x TBS-T and then incubated with secondary antibodies for 1-2 hours at room temperature. Membranes underwent final washes before developing using West Femto Substrate (ThermoFisher 34095) with film exposure or imaged using the Odyssey Li-Cor system. Primary antibodies used: mouse anti-THOC6 (1:1000, Abnova H00079228-A01), rabbit anti-THOC1 (1:2000, Bethyl Laboratories A302-839A), rabbit anti-THOC2 (1:2000, Bethyl Laboratories A303-630A), rabbit anti-THOC5 (1:2000, Bethyl Laboratories A302-120A), mouse anti-ALYREF (1:2000, Sigma Aldrich A9979), rabbit anti-CHTOP (1:2000, Invitrogen PA5-55929), mouse anti-β-actin (1:5000, Abcam ab6276), goat anti-HAPLN1 (1:400, R&D Systems AF2608), rabbit anti-CEMIP (1:2500, Proteintech 50-173-3270), rabbit anti-WNT7A (1:1000, Abcam ab274321), rabbit anti-DKK2 (1:250,ab38594), rabbit anti-TP53 (1:1000, Abcam ab131442), rabbit anti-Flag antibody (1:2500, Proteintech 20543-1-AP). Secondary antibodies used: donkey anti-rabbit HRP-conjugated (1:5000, Cytiva NA9340V), goat anti-mouse HRP-conjugated (1:1000, Invitrogen 32430), IRDye® 680RD goat anti-rabbit IgG (1:5000, Li-Cor Biosciences 926-68071), and IRDye® 800CW goat anti-mouse IgG (1:5000, Li-Cor Biosciences 925-32210).

### **Immunofluorescence and single-molecule fluorescence** in situ **hybridization**

Human NPCs were fixed in 4% paraformaldehyde (PFA) for 20 minutes. Human cortical organoids and mouse embryos were fixed in 4% PFA for 24 hours at 4 °C, cryoprotected in 15% and 30% sucrose in 1x DPBS for 24 hours at 4 °C, then embedded in OCT with quick freezing in −50 °C 2-methylbutane, followed by cryosectioning for immunostaining. Mouse embryos were sectioned at 13 µm and organoids at 16 µm. Samples for immunostaining were incubated for 1 hour with blocking buffer (5% NDS (Jackson ImmunoResearch) 0.1% Triton X-100, 5% BSA) at room temperature, then overnight with primary antibodies diluted in blocking buffer at 4 °C, and for 1–2 hours in secondary dilution at room temperature. Washes performed in PBS. For nuclear staining, samples were incubated at room temperature for 10 minutes in Hoechst or DAPI (1:1000 dilution in PBS) prior to final washes. For EdU labeling detection, the Click-IT EdU imaging kit (Invitrogen C10337) was used according to the manufacturer’s instructions. After incubation with the Click-IT reaction cocktail, sections were blocked and immunostained as described above. Some antibodies required antigen retrieval via incubation in a heated 10 mM sodium citrate solution (95-100 °C) for 20 minutes prior to immunostaining. Primary antibodies used: rabbit anti-KI67 (1:200, Abcam ab16667), rat anti-PH3 (1:250, Abcam ab10543), rabbit anti-cleaved caspase-3 (1:100-1:400, Cell Signaling 9661), mouse anti-N-Cadherin (BD Biosciences 610920), goat anti-DCX (1:400, Santa Cruz Biotechnology C-18, sc-8066), rat anti-CTIP2 (1:500, Abcam ab18465), rabbit anti-PAX6 (1:100, BioLegend PRB-278P), and goat anti-SOX1 (1:100, R&D Biosystems AF3369). AlexaFluor-conjugated secondaries used: donkey anti-mouse 647 (1:400, Invitrogen A31571), donkey anti-rat 555 (1:400, Invitrogen A48270), and donkey anti-rabbit 488 (1:400, Invitrogen A21206).

Embryos and organoids used for RNA Fluorescence in situ Hybridization (FISH) were fixed and cryoprotected as indicated above using RNAse-free PBS. RNAse-zap treatment of sectioning equipment was performed prior to cryosectioning. NPCs for RNA FISH were fixed in RNAse-free 4% PFA and then permeabilized in PBS-TritonX (0.1%) for 15 minutes. Hybridizations were then performed overnight at 37 °C with a final concentration of 2 ng/µl of Cy3-conjugated oligo-dT(30-mer) probe, *MALAT1* (Quasar-670, Stellaris VSMF-2211-5), and/or *MEG3* (Quasar-570, Stellaris VSMF-20346-5). Saline-sodium citrate washes were performed before and after hybridization, followed by nuclear staining with RNAse-free Hoechst-PBS wash (1:1000 dilution) and a final wash in RNAse-free PBS.

Glass covers were mounted onto all slides with Prolong Gold (Molecular Probes S36972) and incubated for 24 hours at room temperature prior to imaging. Imaging was performed with a Nikon A1ss inverted confocal microscope using NIS-Elements Advanced Research software. Image analysis was performed using Fiji (ImageJ2v2.9.0) software^107^. For oligo-dT FISH, Z-series images were taken every 0.2 μm across the entire width of cells for each genotype using the same laser intensity settings and collapsed by max intensity using Z project tool in Fiji for quantification of nuclear and cytoplasmic fractions of poly **A**+ intensity by automated quantitation with CellProfiler (v4.2.1). Hoechst signal was used to segment nuclei and the oligo-dT signal to segment the cell body. Three differentiation replicates per genotype. 3D surface plots were made in Fiji.

### WGA inhibition of nuclear export

Confluent NPCs were incubated with digitonin at 30 μg/mL diluted in DMSO and WGA conjugated to Alexa Fluor 488 (Invitrogen, W11261) at 5 μg/mL diluted in DPBS for 5 minutes^50^. Cells were washed to remove digitonin and WGA only was added to media at 5 μg/mL for 1 hour. Control NPCs were only treated with digitonin. Cells were fixed and prepped for oligo-dT FISH as described above.

### RNA sequencing and bioinformatics analysis

Total RNA was extracted from cultured hNPCs (two biological replicates per genotype) using TRIzol Reagent (Invitrogen 15596026) followed by DNAse column treatment using PureLink RNA extraction kit (Invitrogen 12183018 A). Total RNA from dissected E9.5 mouse forebrain tissue (three biological replicates per genotype) was extracted using Picopure RNA isolation kit (Applied Biosystems KIT0204) according to the manufacturer’s recommendations. hNPC and E9.5 mouse forebrain RNA samples were ribo-depleted followed by 151 bp paired-end sequencing on the Illumina NovaSeq 300 cycle, ~20-30 million reads per sample. Library preparation and sequencing was conducted by the Advanced Genomics Core (AGC) at the University of Michigan. ERCC spike-ins (Invitrogen 4456740) were added for sequencing controls at starting concentrations according to the manufacturer’s instructions.

FASTQ files were trimmed with Cutadapt v4.1 using default parameters^108^. Read quality was assessed by FASTQC v0.11.9^109^. MultiQC v1.7^110^ was used to visualize FASTQC outputs and compare samples. ERCC spike-in FASTA and GTF annotation files were merged with human GRCh38.p13 reference genome FASTA with GTF release 39 or mouse GRCm39 reference genome FASTA with GTF release M28. FASTQ reads were then mapped to merged files using STAR alignment with parameter ‘--outSAMtype BAM SortedByCoordinate’^111^. Count analysis was performed on sorted BAM files using RSEM with paired-end alignment specified^112^. Differential expression analysis was carried out using DESeq2 v1.34.0^113^ in R v4.1.2^114^. ERCC spike-in counts were used to estimate size factors for each sample for DESEq2 analysis. Genes were considered dysregulated if FDR < 0.05 and fold-change > 2 or < −2. Volcano and PCA plots were made using ggplot2 and pcaExplorer packages in R.

Alternative splicing analysis was performed on sorted BAM files using rMATS v4.1.2^115^ with the following parameters: ‘-t paired --readLength 150 --variable-read-length --nthread 4’^55^. AS events were called if FDR < 0.05 and ΔPSI > 10%. Events with less than 5 average reads were filtered out using the MASER package in R^116^. To calculate splice site strength at 5’ and 3’ splice sites in AS transcripts identified by rMATS, maximum entropy modeling was carried out using MaxEntScan^62^. The required input is a 9-mer sequence at 5’ splice sites (3 bases in exon and 6 bases in downstream intron) and a 23-mer at 3’ splice site (20 bases of intron and 3 bases of downstream exon). Scores were plotted in GraphPad Prism (v9.3.1).

DAVID (david.ncifcrf.gov/tools)^117^ and Metascape (metascape.org)^118^ analyzes were performed to identify enriched biological pathways based on Benjamini-Hochberg multiple hypothesis corrections of the *p*-values. To explore evidence for RNA-binding protein motifs at AS junctions, CentriMo Local Motif Enrichment Analysis was performed (MEME Suite 5.5.2). To identify potential transcription factors responsible for expression differences, Gene Set Enrichment Analysis (GSEA v4.2.3) against the MSigDB transcription factor motif gene set (c4.tftv7.5.1.symbols.gmt) and ChIP-X Enrichment Analysis v3 (ChEA3) were performed^119^. Ensembl BioMart tool (http://useast.ensembl.org/biomart) was used to obtain coding sequence length, transcript number per gene, gene type, and sequences for AS events. The GeneOverlap v1.32 R package was used to identify overlapping DE and AS hits between affected genotypes. Primary and candidate syndromic ID genes were obtained from the SysID database (https://www.sysid.dbmr.unibe.ch).

### RT-qPCR and mRNA half-life analysis

Reverse transcription for cDNA synthesis was performed using 1 μg of total RNA with a Superscript III first-strand synthesis kit (Invitrogen 18080051) according to the manufacturer’s instructions. For validation of AS events, standard PCR was performed as described above. Abundances of *THOC6*, *FOS*, *GAPDH*, *MEG3*, *MEG8*, *ESRG*, and *NEAT1* mRNA was determined by quantitative real-time PCR (qPCR) using the Applied Biosystems 7500 system with 7500 Software v2.3 and Radiant Green 2X qPCR Mix Lo-ROX 2X qPCR Mix (Alkali Scientific inc., QS1020) according to manufacturer’s instructions. Cycler parameters used: cDNA activation (1 cycle at 95 °C for 2 minutes), denaturation (40 cycles 95 °C for 5 seconds) and annealing/extension (40 cycles at 60 °C for 30 seconds). The ΔΔCt method was used to analyze data with *GAPDH* as a reference gene. ΔΔCt values obtained by subtracting mean *THOC6*^*+/+*^ ΔCt values for each sample. Data shown represent mean values of three qPCR technical replicates per sample for three biological replicates per genotype (independent differentiations for NPCs). Melt curve analysis was performed on all primers to ensure temperature peaks at ~80–90 °C. *GAPDH* and *FOS* primer sequences were obtained from ref. ^49^. *NEAT1* was obtained from ref. ^74^ and *MEG3* was obtained from ref. ^75^. All others were designed using NCBI primer blast. Primer sequences provided in Table S2.

mRNA stability analysis was performed using transcription inhibition by Actinomycin D (ActD) based on^49^. Human ESCs/iPSCs were passaged into five 12-well plates. Each plate had the following lines: *THOC6*^*+/+*^ (H9 ESCs), *THOC6*^*+/+*^ (AS0041 iPSCs), *THOC6*^W100*/W100*^, *THOC6*^W100*/+^, *THOC6*^*E188K/E188K*^*, THOC6*^*E188K/+*^. Once confluent, ActD was added to the media of all four plates at 10 μg/mL (Sigma-Aldrich A9415). After 30 minutes, media was removed from one plate and 1 mL of TRIzol Reagent was added directly to each well (*t* = 0). Cells were uplifted in TRIzol by pipetting and transferred to a fresh tube. Tubes were immediately frozen in TRIzol at −80 °C. This was repeated every 30 minutes to obtain the following time points 30 minutes post-ActD treatment: *t* = 0.5, 1, 1.5, and 2 hrs. Extractions were performed in batches per time point based on the protocol described above. Standard curve analysis was performed to validate primers (Fig. S1C). This experiment was repeated to capture a longer decay window using the following time points: *t* = 0, 2, 4, 8 (Fig. S1E). Both time-course experiments were repeated in triplicate. Abundances of *THOC6*, *GAPDH*, and *FOS* (positive control for rapid decay) mRNA were determined as described above. ΔΔCt values obtained by subtracting mean *t* = 0 ΔCt for each genotype.

### Quantification and statistical analyses

Statistical significance of all quantifications from microscopy images, western blot images, gel images, and RT-qPCR abundances was tested using a student’s two-tailed *t*-test and data was plotted using GraphPad Prism (v9.3.1) as mean ± SEM or mean ± SD, as specified in figure legends. Simple linear regression was performed in RT-qPCR standard curve analysis, organoid growth curves, and intron retention analysis. Statistical significance of gene overlaps were tested using Fisher’s exact test via GeneOverlap package function testGeneOverlap() in R (v4.2.2). Benjamini-Hochberg multiple hypothesis corrections were performed in pathway enrichment analyzes.

### Reporting summary

Further information on research design is available in the Nature Portfolio Reporting Summary linked to this article.

### Supplementary information

Supplementary Information

Description of Additional Supplementary Files

Supplementary Data 1

Supplementary Data 2

Supplementary Data 3

Reporting Summary

### Source data

Source Data

## Data Availability

Source data are provided with this paper. The raw RNAseq data generated in this study have been deposited in the GEO database under accession code GSE245121 [https://www.ncbi.nlm.nih.gov/geo/query/acc.cgi?acc=GSM7837075]. The clinical data and the processed RNAseq data generated in this study are provided in the Supplementary Information/Source Data file. Genome references used include human GRCh37.p13, human GRCh38.p13 reference, and mouse GRCm39. Databases and datasets used include 1000 Genomes Project phase 311, Genome Aggregate Database (gnomADv3), National Heart, Lung, and Blood Institute Exome Sequencing Project Exome Variant Server (ESP6500SI-V2), SysID database (https://www.sysid.dbmr.unibe.ch), and the MSigDB transcription factor motif gene set (c4.tftv7.5.1.symbols.gmt). Further information and requests for resources and reagents should be directed to and will be fulfilled by the lead contact, Stephanie L. Bielas (sbielas@umich.edu). Source data are provided with this paper.
