# Supplementary material for: TREX tetramer disruption alters RNA processing necessary for corticogenesis in THOC6 Intellectual Disability Syndrome
Source: Nat Commun. 2024 Feb 22;15:1640. doi: 10.1038/s41467-024-45948-y (PMC10884030; doi:10.1038/s41467-024-45948-y)
Supplement: Supplementary file 1 — Supplementary Information [file 41467_2024_45948_MOESM1_ESM.pdf]

SUPPLEMENTARY INFORMATION
for

TREX tetramer disruption alters RNA processing necessary for corticogenesis in THOC6 Intellectual Disability Syndrome

Elizabeth A. Werren[1,2], Geneva R. LaForce[3], Anshika Srivastava[1,4], Delia R. Perillo[1], Shaokun Li[2], Katherine Johnson[2], Safa Baris[5], Brandon Berger[2], Samantha L. Regan[1], Christian D. Pfennig[1], Sonja de Munnik[6], Rolph Pfundt[6], Malavika Hebbar[7], Raúl Jimenez-Heredia[8], Elif Karakoc-Aydiner[5], Ahmet Ozen[5], Jasmin Dmytrus[9], Ana Krolo[8], Ken Corning[10], EJ Prijoles[10], Raymond J. Louie[10], Robert Roger Lebel[11], Thuy-Linh Le[12], Jeanne Amiel[12,13], Christopher T. Gordon[12], Kaan Boztug[8,9,14,15], Katta M. Girisha[16], Anju Shukla[16], Stephanie L. Bielas[1,17,18], Ashleigh E. Schaffer[3,18]

[1]Department of Human Genetics, University of Michigan Medical School, Ann Arbor, Michigan, 48109, USA
[2]Advanced Precision Medicine Laboratory, The Jackson Laboratory for Genomic Medicine, Farmington, Connecticut, 06032, USA
[3]Department of Genetics and Genome Sciences, Case Western Reserve University School of Medicine, Cleveland, Ohio, 44106, USA
[4]Department of Medical Genetics, Sanjay Gandhi Postgraduate Institute of Medical Sciences, Lucknow, Uttar Pradesh, 226014, India
[5]Division of Pediatric Allergy and Immunology, School of Medicine, Marmara University, Istanbul Jeffrey Modell Diagnostic and Research Center for Primary Immunodeficiencies, The Isil Berat Barlan Center for Translational Medicine, Istanbul, 34722, Turkey
[6]Department of Human Genetics, Radboud University Medical Centre Nijmegen, Nijmegen, 6524, the Netherlands
[7]Division of Genetic Medicine, Department of Pediatrics, University of Washington, 98195, USA
[8]Ludwig Boltzmann Institute for Rare and Undiagnosed Diseases, Vienna, 1090, Austria
[9]Research Centre for Molecular Medicine of the Austrian Academy of Sciences, Vienna, 1090, Austria
[10]Greenwood Genetic Center, Greenwood, South Carolina, 29646, USA
[11]Section of Medical Genetics, SUNY Upstate Medical University, Syracuse, NY, 13210, USA
[12]Imagine Institute, INSERM U1163, Paris Cité University, Paris, 75015, France
[13]Service de Médecine Génomique des Maladies Rares, Hôpital Necker-Enfants Malades, AP-HP, Paris, 75015, France
[14]Department of Pediatrics and Adolescent Medicine, Medical University of Vienna, Vienna, 1090, Austria
[15]St. Anna Children's Hospital and Children's Cancer Research Institute, Department of Pediatrics, Medical University of Vienna, Vienna, 1090, Austria
[16]Department of Medical Genetics, Kasturba Medical College, Manipal, Manipal Academy of Higher Education, Manipal, 576104, India
[17]Department of Pediatrics, University of Michigan Medical School, Ann Arbor, Michigan, 48109, USA
[18]These authors jointly supervised this work

This file includes the following supplementary information:

- Supplementary Figures S1-S7 and legends (pp. 3-16)
- Supplementary Tables S1-S2 and legends (pp. 17-20)
- Legends for Supplementary Data 1-3 (p. 21)

FIGURE S1

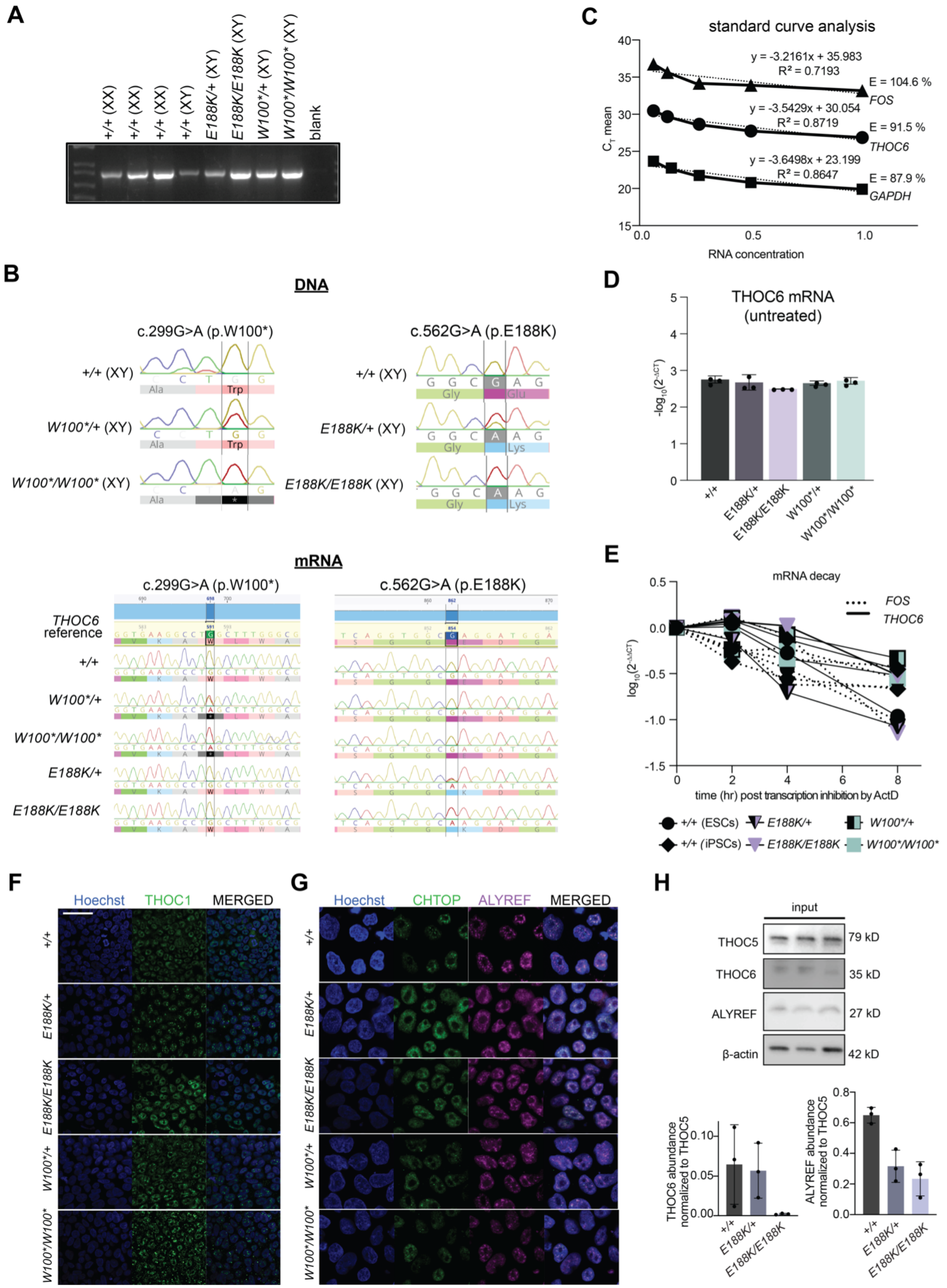

A
+/+ (XX)
+/+ (XX)
+/+ (XX)
+/+ (XY)
E188K/+ (XY)
E188K/E188K (XY)
W100*/+ (XY)
W100*/W100* (XY)
blank
B
DNA
c.299G>A (p.W100*)
c.562G>A (p.E188K)
+/+ (XY)
W100*/+ (XY)
W100*/W100* (XY)
E188K/+ (XY)
E188K/E188K (XY)
mRNA
THOC6 reference
+/+
W100*/+
W100*/W100*
E188K/+
E188K/E188K
C
standard curve analysis
y = -3.2161x + 35.983
R² = 0.7193
E = 104.6 %
FOS
y = -3.5429x + 30.054
R² = 0.8719
E = 91.5 %
THOC6
y = -3.6498x + 23.199
R² = 0.8647
E = 87.9 %
GAPDH
CT mean
RNA concentration
D
THOC6 mRNA (untreated)
-log10(2^-ΔCT)
+/+
E188K/+
E188K/E188K
W100*/+
W100*/W100*
E
mRNA decay
FOS
THOC6
log10(2^-ΔΔCT)
time (hr) post transcription inhibition by ActD
+/+ (ESCs)
+/+ (iPSCs)
E188K/+
E188K/E188K
W100*/+
W100*/W100*
F
Hoechst
THOC1
MERGED
G
Hoechst
CHTOP
ALYREF
MERGED
H
input
THOC5
THOC6
ALYREF
β-actin
79 kD
35 kD
27 kD
42 kD
THOC6 abundance normalized to THOC5
ALYREF abundance normalized to THOC5

Figure S1. Genetic mechanism of biallelic pathogenic *THOC6* variants*. See also Figures 1 and 2.* Confirmation of genotypes in extracted gDNA from human ESC/iPSC lines by (A) PCR and (B) Sanger sequencing (top) as well as in cDNA used for qPCR experiments (bottom). (C) Standard curve analysis using 5 two-fold serial dilutions of control cDNA to confirm primer quality for *FOS, THOC6,* and *GAPDH* for mRNA stability assay*.* (D) THOC6 mRNA expression assessed by RT-qPCR across genotypes. This experiment was repeated independently three times with similar results. (E) mRNA decay curve for extended time frame capturing *THOC6* and *FOS* RNA decay after 2.5 hrs following Actinomycin D treatment. Values were not normalized to *GAPDH* because control transcripts are also degraded after 2.5 hrs of transcription inhibition. This experiment was repeated independently three times with similar results. Immunostaining to assess subcellular localization of THO/TREX complex members THOC1 (F), and CHTOP and ALYREF (G) in human ESC/iPSCs with biallelic pathogenic *THOC6* variants compared to heterozygous and wildtype unaffected controls. (H) THOC5 co-IP input and quantifications of THOC6 and ALYREF normalized to THOC5. *N* = 3 biological replicates of iPSCs/NPCs. Data represented as mean ±SEM. This experiment was repeated independently three times with similar results. Source data are provided as a Source Data file.

FIGURE S2

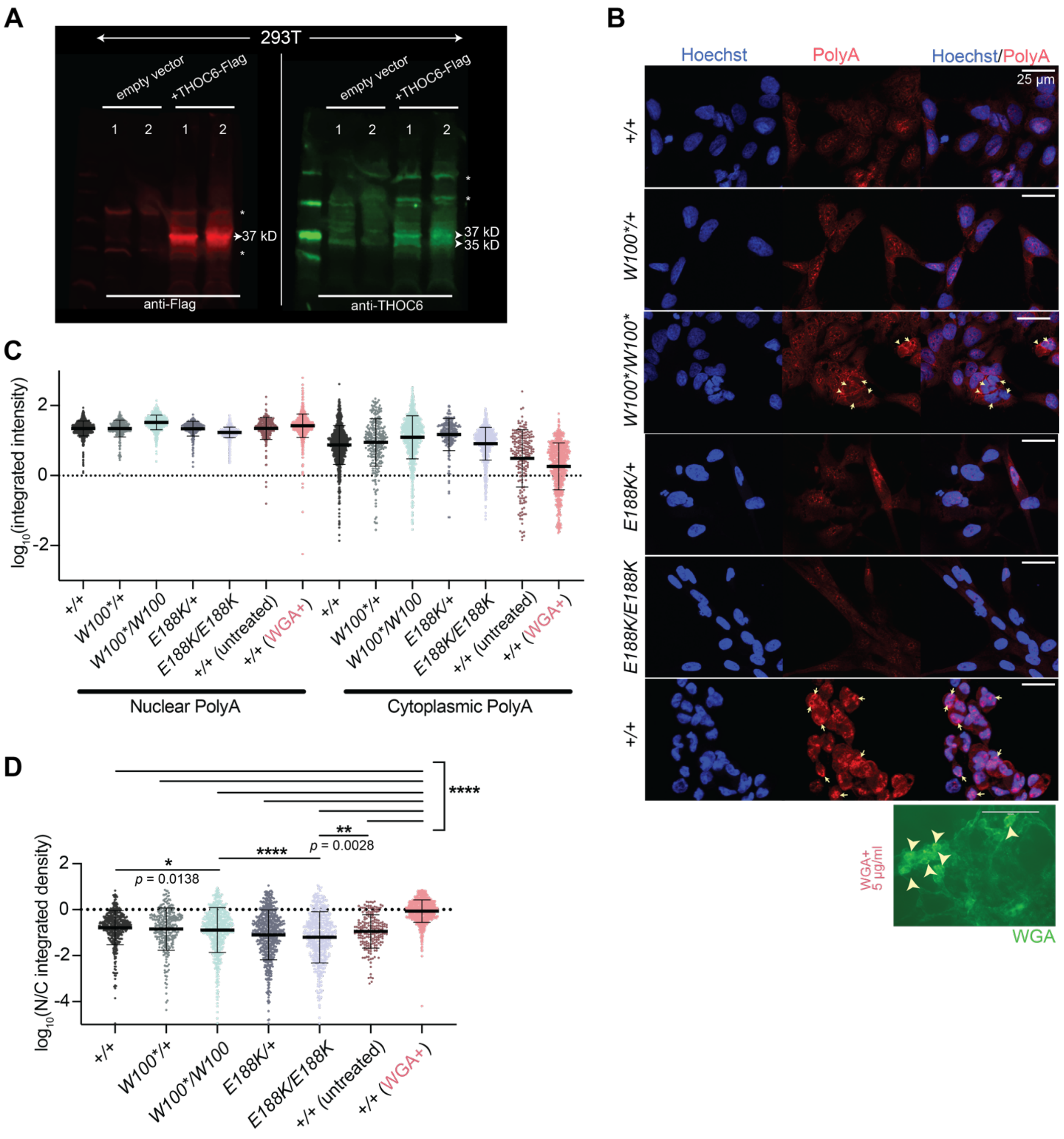

Figure S2. Characterization of TREX composition and bulk mRNA export across genotypes. *Related to Figures 2-5.* (A) Full western blot showing transfection of THOC6-Flag expression vector in two replicates of 293T cells with two empty vector replicates probed with anti-FLAG (red) and anti-THOC6 (green), confirming anti-THOC6

antibody specificity. (B) Oligo-dT FISH Z-collapsed confocal images (40x magnification) of NPCs differentiated from iPSCs with the following genotypes: $THOC6^{+/+}$, $THOC6^{W100*/W100*}$, $THOC6^{W100*/+}$, $THOC6^{E188K/E188K}$, $THOC6^{E188K/+}$. Arrows point to cells showing extreme signal differences. As a positive control for impaired nuclear export resulting in aberrant accumulation of transcripts in the nucleus, $THOC6^{+/+}$ were treated with WGA at 5 µg/ml which acts to block the nuclear pore complex (bottom). Scale bar: 25 µm. (C) Intensity quantifications from 200-700 cells per genotype from three replicates were performed using an automated CellProfiler (v4.2.1) pipeline[129] that measures poly(A)+ signal in nuclear and cytoplasmic fractions. Variability in poly(A)+ signal intensity observed across genotypes is likely due to slight technical variation in the assay across replicate slides. *N* = 300-600 cells across 3 independent biological replicates per genotype. (D) Ratios of nuclear to cytoplasmic poly(A)+ showing minimal differences in bulk export across genotypes relative to WGA+ positive control. *N* = 300-600 cells across 3 independent biological replicates per genotype. Significance obtained by two-tailed, unpaired *t* test. **** indicates $p = < 0.0001$. This experiment was repeated independently three times with similar results.

FIGURE S3

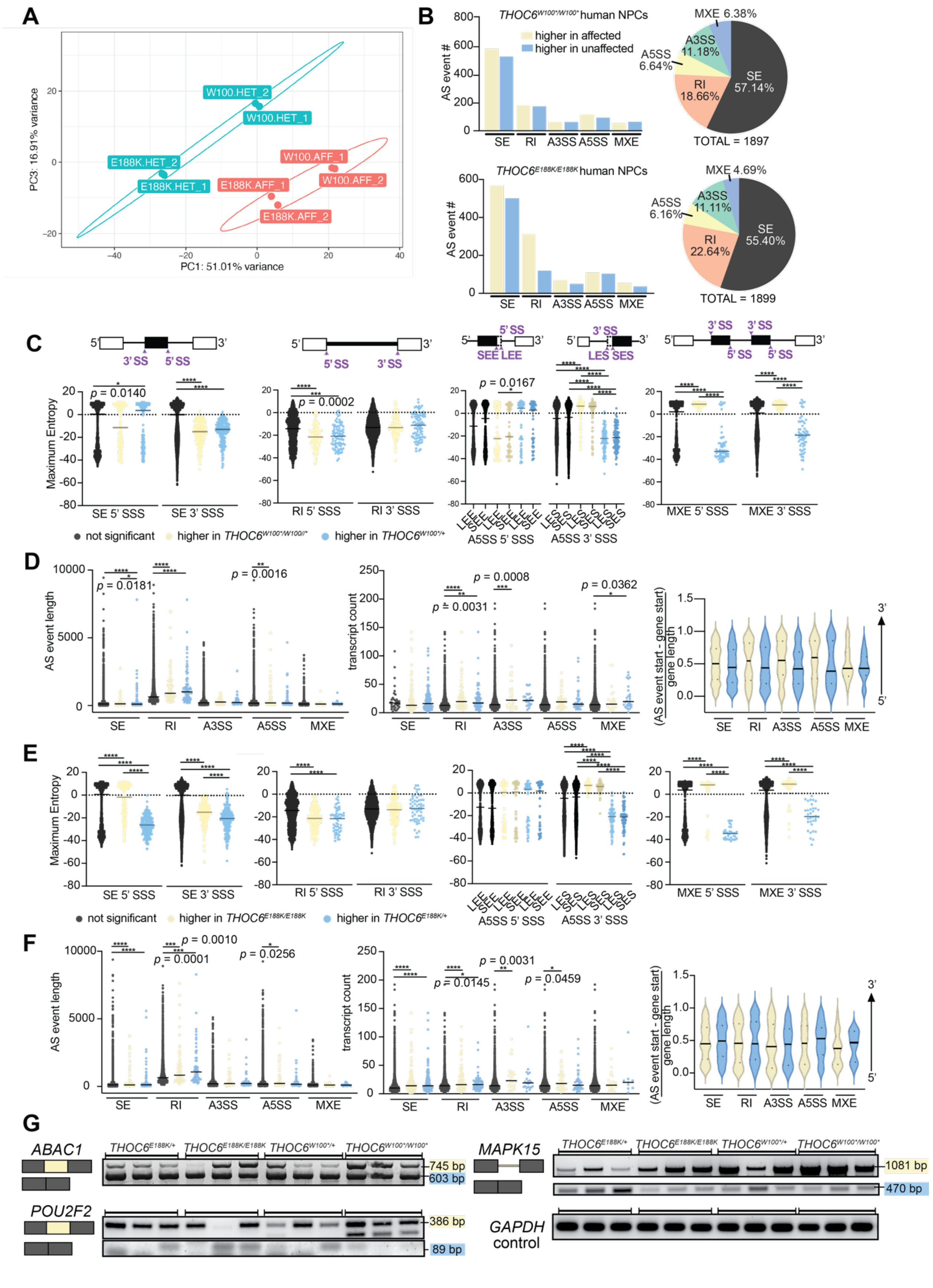
A
PC1: 51.01% variance
PC3: 16.91% variance
W100.HET_2
W100.HET_1
E188K.HET_2
E188K.HET_1
W100.AFF_1
W100.AFF_2
E188K.AFF_1
E188K.AFF_2
B
higher in affected
higher in unaffected
AS event #
SE RI A3SS A5SS MXE
TOTAL = 1897
TOTAL = 1899
C
Maximum Entropy
SE 5' SSS SE 3' SSS RI 5' SSS RI 3' SSS A5SS 5' SSS A5SS 3' SSS MXE 5' SSS MXE 3' SSS
not significant
D
AS event length
transcript count
(AS event start - gene start) gene length
E
F
G
ABAC1
POU2F2
MAPK15
GAPDH control
745 bp
603 bp
386 bp
89 bp
1081 bp
470 bp

Figure S3. Characterization of alternative splicing events in *THOC6* affected hNPCs. *See also Figure 3.* (A) PCA of hNPCs RNAseq samples showing separation by condition: unaffected in teal (E188K_HET, W100_HET) and affected in coral (E188K_AFF, W100_AFF). The remaining analyses were performed on two biological replicates, three technical replicates, per genotype. (B) rMATS summary analysis on $THOC6^{W100*/W100*}$ versus $THOC6^{W100*/+}$ hNPCs (top) and $THOC6^{E188K/E188K}$ versus $THOC6^{E188K/+}$ hNPCs (bottom). Differences in splicing patterns were comparable in both $THOC6^{E188K/E188K}$ versus $THOC6^{E188K/+}$ and $THOC6^{E188K/E188K}$ versus $THOC6^{W100*/+}$ comparisons, as well as compared to $THOC6^{W100*/W100*}$ versus $THOC6^{W100*/+}$ (see Table S5 and Figure 4). Blue, excluded in affected; yellow, included in affected. (C-D) 5' and 3' splice site strengths (SSS), and event length, transcript count, and event position per AS event in $THOC6^{W100*/W100*}$ hNPCs. Long exon start, LES; short exon start, SES; long exon end, LEE; short exon end, SEE. Black, events not significant. Yellow, higher inclusion in affected condition. Blue, higher inclusion in unaffected condition. Significance obtained by two-tailed, unpaired *t* test. **** indicates p = < 0.0001. (E-F) 5' and 3' splice site strengths, and event length, transcript count, and event position per AS event in $THOC6^{E188K/E188K}$ hNPCs. Long exon start, LES; short exon start, SES; long exon end, LEE; short exon end, SEE. Black, events not significant. Yellow, higher inclusion in affected condition. Blue, higher inclusion in unaffected condition. Significance obtained by two-tailed, unpaired *t* test. **** indicates p = < 0.0001. (G) RT-PCR gels across three biological hNPC replicates per genotype for *ABAC1*, *POU2F2*, and *MAPK15* AS events and *GAPDH* loading control. Some variation across replicates

was noted. This experiment was repeated twice with similar results. Source data are provided as a Source Data file.

FIGURE S4

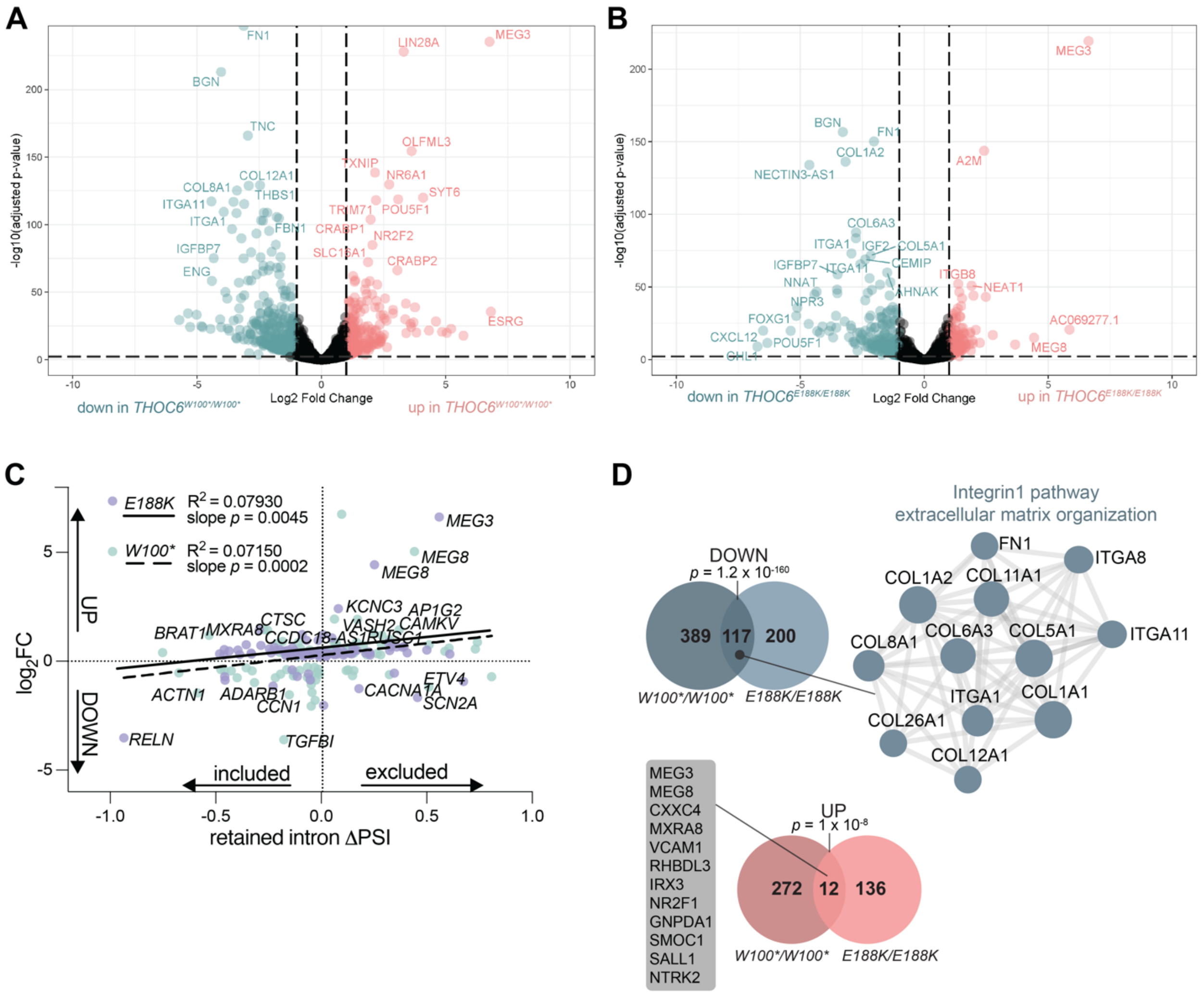

Figure S4. Differential expression analysis in affected hNPCs. *See also Figure 4.* Volcano plot of differential expression in *THOC6*$^{W100*/W100*}$ (A) and *THOC6*$^{E188K/E188K}$ (B) NPCs relative to *THOC6*$^{W100*/+}$ controls. Data represent analysis of two biological replicates per genotype. The later comparison was chosen following observation of

upregulated skeletal muscle genes in *THOC6*$^{E188K/+}$ hNPC replicates, indicating issues during the differentiation of this line. Teal represents downregulated in affected cells; coral represents upregulated in affected cells. Log_2foldchange cut-offs at < -1 and > 1. (C) Linear regression analysis of log_2foldchange and percent transcripts spliced in (deltaPSI) for significant retained intron events in affected cells. Purple dots indicate *THOC6*$^{E188K/E188K}$ hits and green dots indicate *THOC6*$^{W100*/W100*}$. Best fit line, R^2, and slope p-value for *THOC6*$^{E188K/E188K}$ (solid line) and *THOC6*$^{W100*/W100*}$ (dotted line). (D) Gene overlap of *THOC6*$^{W100*/W100*}$ and *THOC6*$^{E188K/E188K}$ downregulated (left, blue) and upregulated (right, red) genes. Metascape protein-protein network enrichment analysis identifies integrin1 pathway and extracellular matrix modules enriched among genes downregulated in both genotypes. Overlap significance tested by one-sided Fisher's exact test.

FIGURE S5

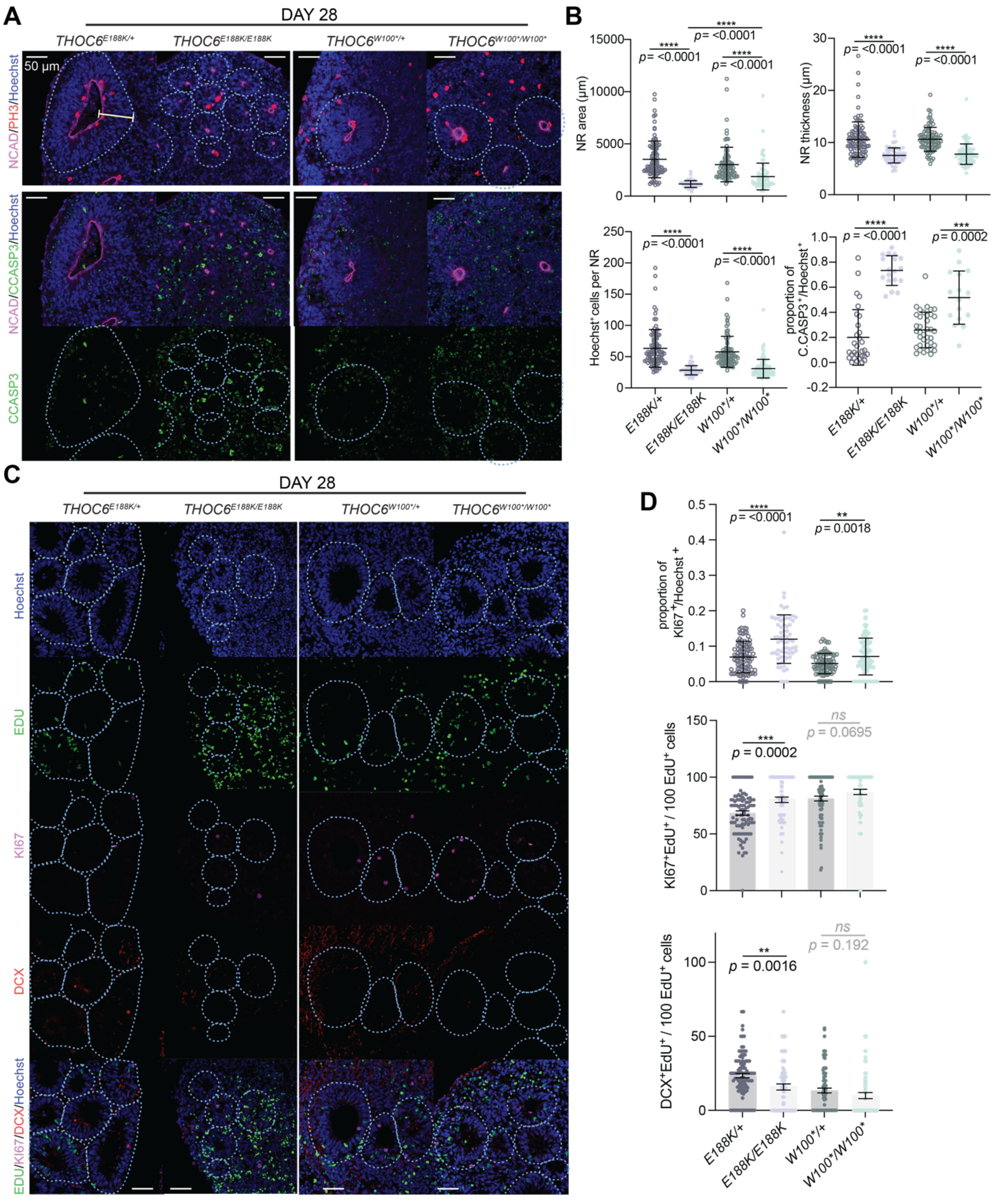

Figure S5. Modeling of *THOC6* variant pathogenesis in human cerebral organoids.

See also Figure 5. (A) Immunostaining of PH3, N-Cadherin, C.CASP3, and Hoechst in

day 28 human cerebral organoids differentiated from *THOC6*$^{E188K/+}$, *THOC6*$^{E188K/E188K}$, *THOC6*$^{W100*/+}$, and *THOC6*$^{W100*/W100*}$ iPSCs highlighting differences in neural rosette morphology. 40x magnification; Scale bar: 50 µm. (B) Quantifications by genotype of area, thickness, Hoechst+ cells, and C.CASP3+ cells per NR. NR (organoid) number analyzed across one differentiation replicate per genotype: *THOC6*$^{E188K/+}$ *n* = 30 (5); *THOC6*$^{E188K/E188K}$ *n* = 18 (5); *THOC6*$^{W100*/+}$ *n* = 37 (10); *THOC6*$^{W100*/W100*}$ *n* = 16 (5). (C) Immunostaining of EDU, KI67, DCX to assess timing of differentiation in day 28 organoids with quantifications by genotype. (D) Quantifications for (C); Genotypes: *THOC6*$^{E188K/+}$ (dark grey purple), *THOC6*$^{E188K/E188K}$ (purple), *THOC6*$^{W100*/W100*}$ (dark grey green), *THOC6*$^{W100*/W100}$ (green). NR (organoid) number analyzed across three differentiation replicates per genotype: *THOC6*$^{E188K/+}$ *n* = 100 (34); *THOC6*$^{E188K/E188K}$ *n* = 68 (25); *THOC6*$^{W100*/+}$ *n* = 87 (53); *THOC6*$^{W100*/W100*}$ *n* = 89 (42). Significance, two-tailed unpaired *t* test. Data shown as mean ±SEM.

FIGURE S6

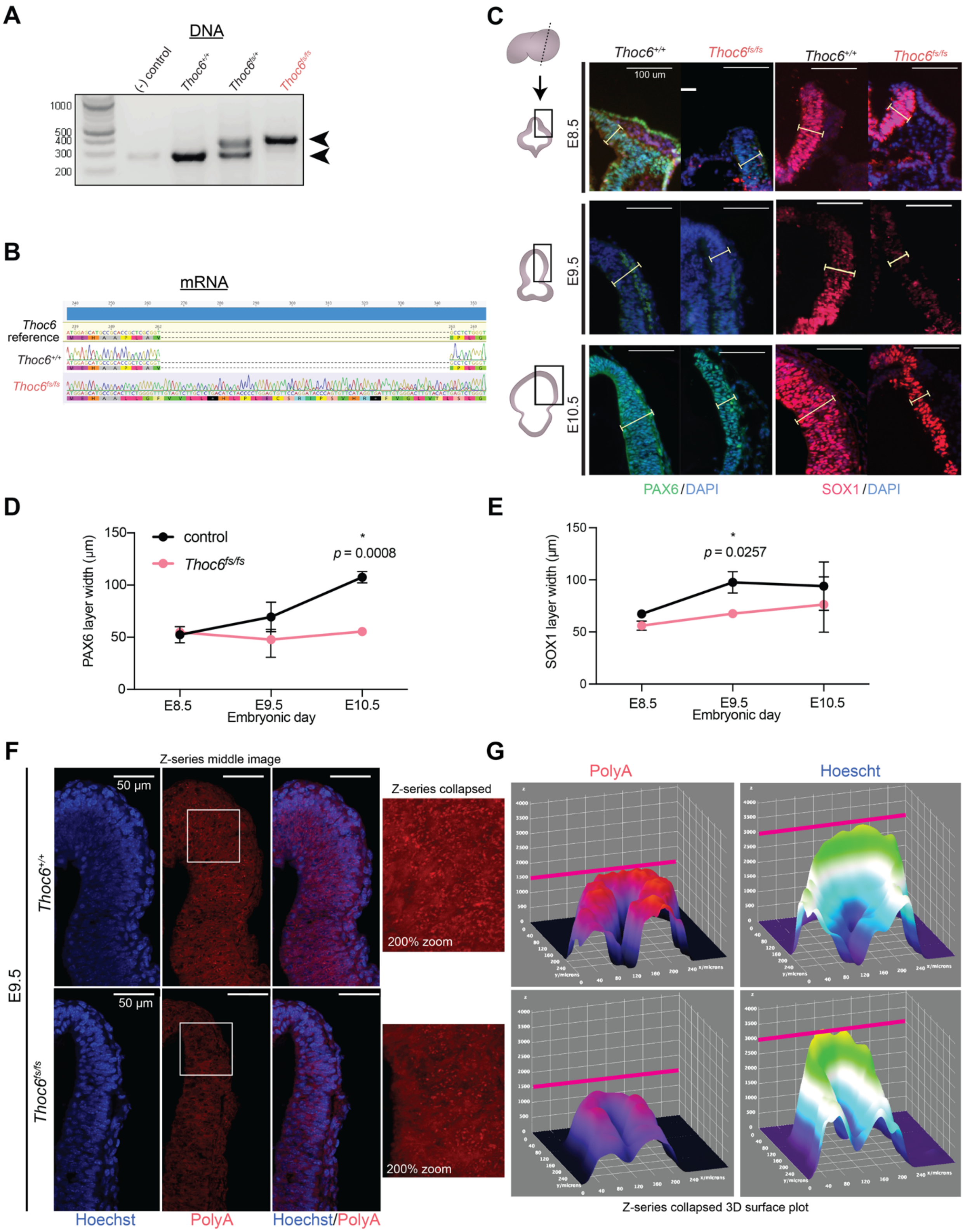
A
DNA
(-) control
Thoc6+/+
Thoc6fs/+
Thoc6fs/fs
1000
500
400
300
200
B
mRNA
Thoc6 reference
Thoc6+/+
Thoc6fs/fs
C
Thoc6+/+
Thoc6fs/fs
100 um
E8.5
E9.5
E10.5
PAX6/DAPI
SOX1/DAPI
D
control
Thoc6fs/fs
p = 0.0008
PAX6 layer width (µm)
Embryonic day
E8.5
E9.5
E10.5
E
p = 0.0257
SOX1 layer width (µm)
Embryonic day
F
Z-series middle image
Z-series collapsed
50 µm
200% zoom
E9.5
Hoechst
PolyA
Hoechst/PolyA
G
PolyA
Hoescht
Z-series collapsed 3D surface plot

Figure S6. Generation of *Thoc6*$^{fs/fs}$ mouse model. *See also Figure 6.* (A) Gel of allele-specific PCR confirming *Thoc6* frameshift alleles from extracted DNA. (B) Sanger sequencing confirmation of insertion event that leads to a premature termination codon in cDNA from *Thoc6*$^{fs/fs}$ mice relative to wildtype control. (C) Immunostaining of the neural plate and neural tube in *Thoc6*$^{fs/fs}$ mice relative to *Thoc6*$^{+/+}$ control littermates at E8.5-10.5 with layer width quantifications for Pax6 and Sox1 (D and E, respectively). Pink represents *Thoc6*$^{fs/fs}$; black represents *Thoc6*$^{+/+}$. Significance, two-sided paired Student's *t* test. *n.s.*, not significant. Scale bar: 100 µm. (F) Oligo-dT FISH of E9.5 *Thoc6*$^{+/+}$ and *Thoc6*$^{fs/fs}$ mouse neuroepithelium; Z-series middle image (left). Scale bar: 50 µm. White box represents the area zoomed in on the right (200% zoom of poly(A)+ signal in Z-series collapsed image by maximum intensity). (G) 3D surface plot for Z-series collapsed image of poly(A)+ intensity (left) and Hoechst intensity (right) in *Thoc6*$^{+/+}$ E9.5 neuroepithelium (top) and *Thoc6*$^{fs/fs}$ E9.5 neuroepithelium (bottom). This experiment was repeated three independent times with similar results.

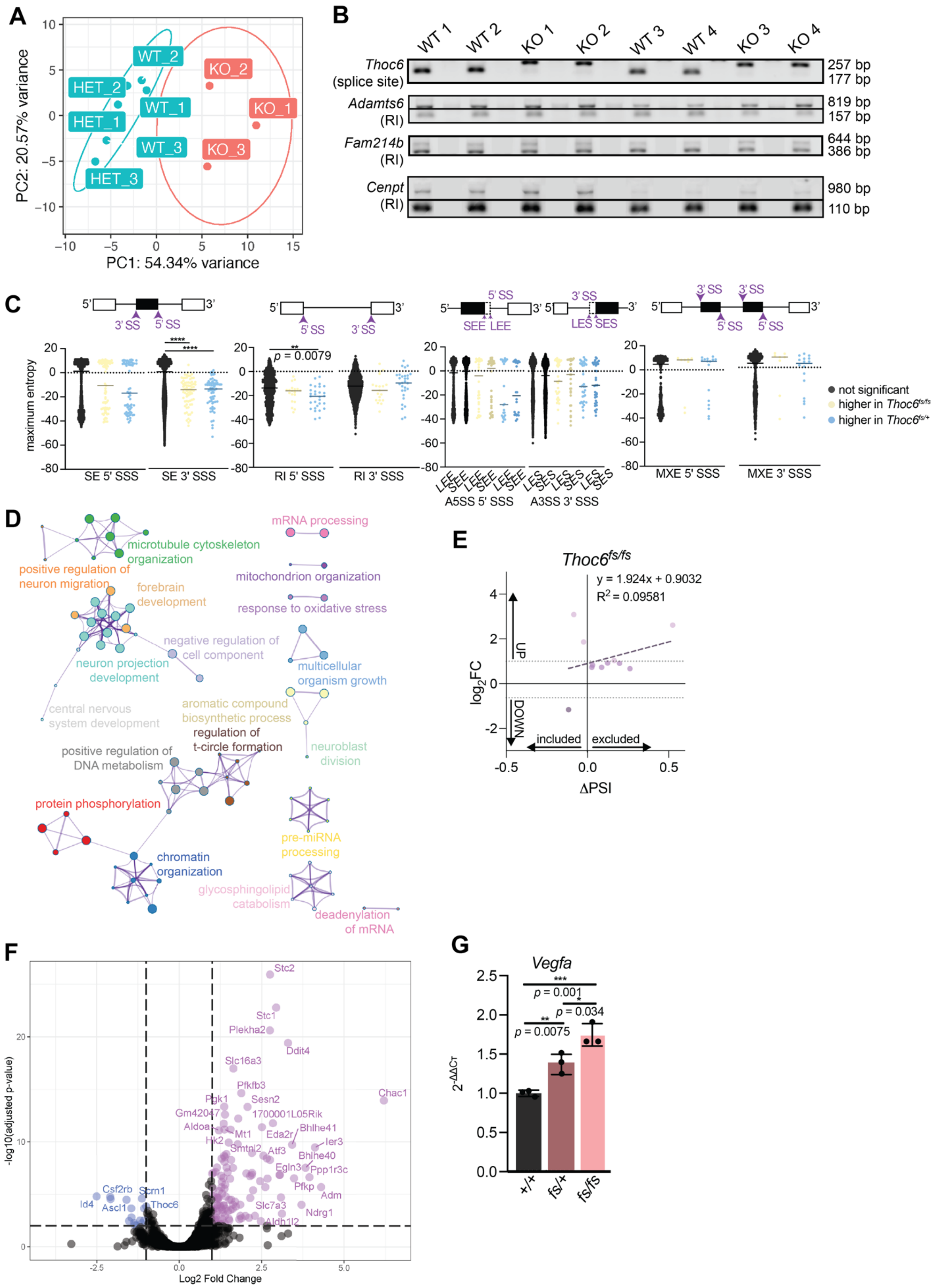

A
B
WT 1
WT 2
KO 1
KO 2
WT 3
WT 4
KO 3
KO 4
Thoc6 (splice site)
Adamts6 (RI)
Fam214b (RI)
Cenpt (RI)
257 bp
177 bp
819 bp
157 bp
644 bp
386 bp
980 bp
110 bp
PC1: 54.34% variance
PC2: 20.57% variance
C
maximum entropy
not significant
higher in Thoc6fs/fs
higher in Thoc6fs/+
SE 5' SSS
SE 3' SSS
RI 5' SSS
RI 3' SSS
A5SS 5' SSS
A3SS 3' SSS
MXE 5' SSS
MXE 3' SSS
p = 0.0079
D
mRNA processing
microtubule cytoskeleton organization
positive regulation of neuron migration
mitochondrion organization
forebrain development
response to oxidative stress
negative regulation of cell component
neuron projection development
multicellular organism growth
central nervous system development
aromatic compound biosynthetic process
regulation of t-circle formation
neuroblast division
positive regulation of DNA metabolism
protein phosphorylation
pre-miRNA processing
chromatin organization
glycosphingolipid catabolism
deadenylation of mRNA
E
Thoc6fs/fs
y = 1.924x + 0.9032
R2 = 0.09581
UP
DOWN
included
excluded
log2FC
ΔPSI
F
-log10(adjusted p-value)
Log2 Fold Change
Stc2
Stc1
Plekha2
Ddit4
Slc16a3
Pfkfb3
Pgk1
Sesn2
Chac1
Gm42047
1700001L05Rik
Aldoa
Bhlhe41
Mt1
Eda2r
Hk2
Ier3
Smtnl2
Atf3
Bhlhe40
Egln3
Ppp1r3c
Pfkp
Adm
Csf2rb
Scrn1
Id4
Ascl1
Thoc6
Slc7a3
Ndrg1
Aldh1l2
G
Vegfa
p = 0.001
p = 0.034
p = 0.0075
+/+
fs/+
fs/fs

FIGURE S7

Figure S7. Characterization of mRNA processing defects in *Thoc6$^{fs/fs}$* mouse E9.5 forebrain. *See also Figure 7.* (A) PCA of mouse E9.5 forebrain RNAseq samples showing separation by genotype (wildtype (WT, teal), heterozygous (HET, teal), homozygous for frameshift (KO, coral). *N* = three biological replicates per genotype. (B) RT-PCR gels across four biological replicates per genotype in two litters showing *Thoc6*, *Admts6*, *Fam214b*, and *Cenpt* AS events. (C) 5’ and 3’ splice site strengths (SSS) across AS events in *Thoc6$^{fs/fs}$* mouse E9.5 forebrain*.* Blue, excluded in affected; yellow, included in affected. Long exon start, LES; short exon start, SES; long exon end, LEE; short exon end, SEE. Significance obtained by two-tailed, unpaired *t* test. **** indicates p = < 0.0001. (D) Metascape visualization of enriched biological categories among AS genes in *Thoc6$^{fs/fs}$* with FDR < 0.05, ΔPSI < -0.1 or > 0.1, and average read coverage > 5. (E) Linear regression analysis of $\log_2$foldchange and percent transcripts spliced in (deltaPSI) for significant retained intron events in *Thoc6$^{fs/fs}$* cells. Log_2foldchange cut-offs at < -1 and > 1. Best fit line and R^2 value on graph. (F) Volcano plot of differential expression analysis in *Thoc6$^{fs/fs}$* relative to *Thoc6$^{+/+}$* control. *N* = 3 biological replicates per genotype. Blue, downregulated in *Thoc6$^{fs/fs}$*; purple, upregulated in *Thoc6$^{fs/fs}$*. (G) qPCR relative abundance for mouse E9.5 forebrain (relative to wildtype) for *Vegfa. N* = 3 experimental replicates of two biological replicates per genotype. Data shown as mean ±SEM. Significance, two-tailed unpaired *t* test. Source data are provided as a Source Data file.

Table S1. Clinical descriptions of individuals with TIDS in present study.

Individual	Clinical description	Genetic diagnosis	Variant Interpretation[a]
P1 (1:II:1)	P1 is from the Netherlands and of European ancestry. P1 was twelve months old at provided evaluation. The pregnancy was complicated by intrauterine growth restriction at thirty weeks of gestation. P1 was born via a spontaneous vaginal delivery after 38 weeks and 3 days of gestation with a birth weight was 2.252 kg, and a length of 47 cm. Feeding problems were noted after birth. Gastroesophageal reflux disease was diagnosed requiring nasogastric tube feeding intervention. Growth parameters at four months of age included a length of 58 cm (<3rd centile), with a weight of 4.275 kg (<3rd centile) and an occipitofrontal circumference (OFC) of 38 cm (<3rd centile). Dysmorphic features include plagiocephaly, upslanting/narrow palpebral fissures, a straight nose with a broad nasal base, a smooth philtrum with a thin upper lip, and protruding ears with large, upturned earlobes (Figure 1B). A renal ultrasound revealed unilateral renal agenesis, though cardiac abnormalities were not detected by ultrasound evaluation. Marked signs of developmental delay at 12 months. Motor delays characterized by deficits in coordination required for prolonged prone positioning on the abdomen and inability to sit unassisted.	Karyotyping results were normal. Trio WES (mother/father/proband) was performed and identified two *THOC6* variants as high priority candidates for the clinical presentation observed for P1. A previously described missense variant was maternally inherited and a novel truncating *THOC6* variant was paternally inherited: c.569G>A, (p.G190E) and c.139C>T, (p.Q47*) (Figure 1A). The c.139C>T, (p.Q47*) variant was not found in the gnomAD Browser (v2.1.1) and was submitted to ClinVar (https://www.ncbi.nlm.nih.gov/clinvar/; Submission ID: SUB5265728). The parents of P1 were heterozygous for the identified variants, consistent with a recessive mode of inheritance.	p.G190E: pathogenic (PM2, PM3, PS3, PP3);<br><br>p.Q47*: pathogenic (PVS1, PM2, PM3)
P2 (2:III:2)	P2 is the second-born child of consanguineous parents of Turkish ancestry, three years old at provided evaluation (Figure 1A). P2 was born full-term (41 weeks of gestation) with a birth weight of 2.7 kg (10th centile) and an OFC of 32.5 cm (<3rd centile). P2 exhibited global developmental delay, feeding problems, and persistent vomiting in the first few postnatal months, consistent with features of gastroesophageal reflux disease. Use of a nasoduodenal feeding tube supported slow, yet persistent weight gain. Physical examination revealed microcephaly and atypical facial features of epicanthus, long nose with low hanging columella, and upslanting palpebral fissures (Figure 1B). Cranial MRI revealed corpus callosum hypoplasia (Figure 1C). Frequent upper respiratory infections occurred one to three times per month. Echocardiogram demonstrated peripheral pulmonary stenosis and patent foramen ovale. Renal anatomy was unremarkable. At 3 years of age, weight was 13 kg (3rd centile), height was 95 cm (3rd centile), and OFC was 45 cm (<3rd centile). Severe intellectual disability was noted with delays in speech and communication requiring special rehabilitation intervention. Motor delays were observed. At time of evaluation, P2 walked with an unsteady gait. While hearing and vision are normal, strabismus was observed. P2 has a high number of dental caries, in line with previously described BBIS dental anomalies.	Karyotyping results were normal. WES genetic testing identified a novel biallelic truncating variant c.299G>A, (p.W100*) in exon 4 of *THOC6* in P2. At the time of identification, this variant was not reported and not observed in the gnomAD Browser (v2.1.1) and was submitted to ClinVar (https://www.ncbi.nlm.nih.gov/clinvar/; Submission ID: SUB5265724). Heterozygosity of the *THOC6* variant was confirmed by Sanger sequencing in the mother, as well as in an unaffected sibling.	p.W100*: pathogenic (PVS1, PM2, PM3)
P3 (3:III:1)	P3 is from the United States of European ancestry, eleven years old at provided evaluation. P3 was evaluated for multiple congenital anomalies during the newborn period. Microcephaly, deep set eyes, mild epicanthal folds, upslanting palpebral fissures were described during examination in infancy; prominent antihelices of the ears, broad nasal bridge, mild depression of the right nostril with evidence of right cleft lip repair, nasal columella extending below nares, and short philtrum were noted (Figure 1B). Clinical history at age eleven was significant for cleft lip, bifid uvula, ankyloglossia, horseshoe kidney, imperforate anus, developmental delay, autism, and partial complex seizures (onset at 11 years). Developmentally, receptive	Karyotype and FISH analysis for 22q11.2 were normal (Oxford Gene Technology Syndrome). Chromosome microarray (Affymetrix CytoScan Dx) identified a maternally inherited deletion on 5q21.1, the inheritance of which is inconsistent with genetic basis of TIDS clinical features. WES analysis of P3 identified three previously described homozygous missense variants in *cis* in *THOC6* c.[298T>A;700G>C;824G>A], (p.[W100R;V234L;G275D]). This	p.W100R: likely pathogenic (PM2, PM3)<br><br>p.V234L: likely pathogenic (PM2, PM3, PP5)<br><br>p.G275D: pathogenic

	language skills were noted to be better than expressive language that was restricted to sign language, some words, and gesturing. Assistive technology was used to supplement this deficit. Reading and math skills at age eleven were measured to be equivalent to first grade level. On physical examination, both OFC and weight were less than the 2nd centile, and height was at the 5th centile.	haplotype was previously reported in three other individuals with TIDS from unrelated families. In all cases, no consanguinity was reported. The parents of P3 are heterozygous for the identified variants, indicating biparental inheritance of the *THOC6* variants.	(PM2, PM3, PS3)
P4.1 (4:IV:1)	P4.1 was evaluated at thirteen years of age and was the first-born child of third-degree consanguineous parents from Southern India (Figure 1A). P4.1 weighed 2.75 kg at birth and had complaints of repeated lower respiratory tract infections since the newborn period. Developmental delay, predominantly cognitive, was noted. P4.1 crawled at nine months, sat at one year, and walked at two years. P4.1 spoke bisyllables only at last examination and exhibited friendly behavior. OFC was 44.5 cm (<3rd centile, -7 SD) and height was 122 cm (normal). Facial dysmorphism included upslanting palpebral fissures, epicanthal folds, microcornea, long nose with overhanging columella, thick vermillion borders, and crowded teeth (Figure 1B). Upon neurological examination, P4.1 had contractures at the ankle, normal to increased tone, and normal deep tendon reflexes. Vision and hearing were normal. Brain imaging results were also normal.	Karyotyping results were normal for P4.1 and P4.2. The biallelic missense variant c.824G>A, (p.G275D) in exon 12 of *THOC6* was identified in P4.1 and P4.2 by WES. Variant p.G275D has previously been observed in individuals with TIDS (ClinVar Accession ID: SCV000741884.1). The parents were heterozygous for the identified variants, as confirmed by Sanger sequencing and consistent with recessive mode of inheritance (Figure 1A).	p.G275D: pathogenic (PM2, PM3, PS3)
P4.2 (4:IV:2)	P4.2 was the younger sibling of P4.1 (Figure 1A). P4.2 was eight years old upon examination and presented with global developmental delay. P4.2 achieved head control at five months of age, sat with support at one year, and stood with and without support at one and two years, respectively. At last examination, P4.2 followed simple commands. Parents also reported nocturnal enuresis. OFC was 44.5 cm (<3rd centile, -6 SD), height was 104 cm, and weight was 12 kg. Epicanthal folds, microretrognathia, cleft palate, and U-shaped uvula were noted (Figure 1B). Vision and hearing were normal.	[see above]	p.G275D: pathogenic (PM2, PM3, PS3)
P5 (5:II:3)	P5 was born to consanguineous parents of Moroccan ancestry. P5 had a healthy dizygotic twin as well as healthy older and younger siblings. P5 was born at 37 weeks of gestation with birth parameters of -2 SD (birth weight of 2.08 kg, birth length of 44 cm, and OFC of 31 cm). P5 presented short-segment Hirschsprung disease, submucous cleft palate, and unilateral choanal stenosis that was surgically repaired at 18 months. P5 had delayed psychomotor development and all growth parameters remained at -2 SD. P5 was shy with nasal speech limited to short sentences. At 10 years of age, P5 had a long narrow face, arched eyebrows, convergent strabismus, a tubular nose with a high nasal bridge, short columella, cupid bow-shaped mouth, and normal ears. Cutaneous 2-3 syndactyly on feet and clinodactyly of the 5th fingers were also noted. No ophthalmologic abnormalities were present except for convergent strabismus. Auditory evoked potential (AEP), brain and temporal bones CT-scan, and cardiac ultrasound were normal.	800-bands resolution karyotype showed normal chromosomes on lymphocytes, 46XX, with no 22q11.2 deletion by FISH analysis at the TUPLE1 locus. WES analysis identified biallelic *THOC6* variants c.740G>A, (p.R247Q). This variant was absent in unaffected siblings whereas unaffected parents were heterozygous, consistent with recessive mode of inheritance. Results were confirmed by Sanger sequencing.	p.R247Q: likely pathogenic (PM2, PM3)

P6 (6:IV:1)	P6 was 13 months old at last examination. P6 had an OFC of 42.5 cm (-3/4 SD) that was likely progressive. P6 had dysmorphic facial features. Hypotonicity, no spasticity, and absent tendon reflexes were noted upon neurological examination. P6 had hypoplastic genitalia. MRI revealed atrophy of the cortex and cerebellum as well as ventriculomegaly with a thin corpus callosum. P6 had intermediate delayed psychomotor development.	WES analysis identified biallelic variants of c.562G>A, (p.E188K). Variant was absent in unaffected siblings and unaffected parents were heterozygous for the variant, consistent with recessive mode of inheritance. Results confirmed by Sanger sequencing.	p.E188K is likely pathogenic (PM2, PM3)
P7 (7:V:1)	P7 was reported to have an OFC of -5 SD, epilepsy, and micropenis at last examination.	WES analysis identified biallelic variants of c.299G>A, (p.W100*). Variant was absent in unaffected siblings and unaffected parents were heterozygous, consistent with recessive mode of inheritance. Results confirmed by Sanger sequencing.	p.W100*: pathogenic (PVS1, PM2, PM3)

[a]Clinical classification according to the American College of Medical Genetics and Genomics (ACMG) guidelines and evidence codes

Table S2. PCR, RT-PCR, and RT-qPCR primer sequences for human and mouse samples.

Primer type	ID	forward sequence	reverse sequence	Species
PCR	*THOC6* DNA genotyping	5' CTTCAGGACTTTGGGTGGGA 3'	5' AAAGAACTGTGAGTGGTGCC 3'	human
	Thoc6 DNA genotyping	5' ACGAGAAGAGCCACCATCAG 3'	5' ATCACTTTTCGTGGGCTCAG 3'	mouse
**RT-PCR**	*THOC6* mRNA genotyping (c.299G>A; p.W100*)	5' CCGGTGGTGACTTTCCAAG 3'	5' GTCCATAGTGTGCAACTGACA 3'	human
	THOC6 mRNA genotyping (c.562G>A; p.E188K)	5' TTTGGGCGGAGATGCTCAAG 3'	5' TGCCAAACATCCAATCCAGC 3'	human
	ABCA1 SE	5' CATTCAGATGCACGTCTGCT 3'	5' GCTGGCTGTCAAAGAGGAAC 3'	human
	POU2F2 SE	5' TTCTGCATGTCCTTCACTGC 3'	5' CTTGCTCCAATTCCTGCTGT 3'	human
	MAPK15 RI	5' CCAGACAGCAGAAACCCTGT 3'	5' TTGGGGGTCTCTGACATAGG 3'	human
	Thoc6 splice site	5' GTTCCCATCGTTCGTACTGG 3'	5' GCTTTGAAGGAAGCCAACTG 3'	mouse
	Admts6 RI	5' GTTCCCATCGTTCGTACTGG 3'	5' GCTTTGAAGGAAGCCAACTG 3'	mouse
	Fam214b RI	5' CAGGCTTGAGGCTTCTTCAT 3'	5' GGTTCTTCAGGGGGTAGTCC 3'	mouse
	Cenpt RI	5' TCCTCCAGCACACATGACTC 3'	5' CTGGATGGGCACTTAGCTGT 3'	mouse
**RT-qPCR**	*GAPDH* qPCR	5' TCTTTTGCGTCGCCAGCCGA 3'	5' ACCAGGCGCCCAATACGACC 3'	human
	FOS qPCR	5' GTGGGAATGAAGTTGGCACT 3'	5' CTACCACTCACCCGCAGACT 3'	human
	THOC6 qPCR	5' CGACTGGATGGTCTGTGGAG 3'	5' CCTGGTAGAAGGTGACGTGC 3'	human
	MEG3 qPCR	5' GCATTAAGCCCTGACCTTTG 3'	5' TCCAGTTTGCTAGCAGGTGA 3'	human
	MEG8 qPCR	5' CCTCAGTATCCTGCGAGCTG 3'	5' AAGTCAGACCCAGGCAACAC 3'	human
	ESRG qPCR	5' CAGCCTTGTACCCTGGTCTT 3'	5' ATGCATTGGCTTGTTGCTGA 3'	human
	NEAT1 qPCR	5' GGCAGGTCTAGTTTGGGCAT 3'	5' CCTCATCCCTCCCAGTACCA 3'	human
	TGFB2 qPCR	5' AAGAAGCGTGCTTTGGATGCGG 3'	5' ATGCTCCAGCACAGAAGTTGGC 3'	human
	ID4 qPCR	5' GGACCTGTCCAGCCGCGCC 3'	5' TCAGCGGCACAGAATGCTGTCG 3'	human
	TP53 qPCR	5' CCTCAGCATCTTATCCGAGTGG 3'	5' TGGATGGTGGTACAGTCAGAGC 3'	human
	PAX6 qPCR	5' CTGAGGAATCAGAGAAGACAGGC 3'	5' ATGGAGCCAGATGTGAAGGAGG 3'	human
	CXXC4 qPCR	5' TGCCCGCAGAATCATTCCTCCT 3'	5' ACGCCACAGTTGATGAGCCTCT 3'	human
	WNT7A qPCR	5' AGGAGAAGGCTCACAAATGGGC 3'	5' CGGCAATGATGGCGTAGGTGAA 3'	human
	Ier3 qPCR	5' CCATCTCCACACCATGACTG 3'	5' CTCCGAGGTCAGGTTCAAAG 3'	mouse
	Islr2 qPCR	5' CTGCAAGTCAGAGAGCAGCA 3'	5' AACTGGTGGGCGTACTTGTC 3'	mouse
	Thoc6 qPCR	5' GCAACAATTACGGGCAGATT 3'	5' CAACCCTTGACCTCTCCATC 3'	mouse
	Anxa2 qPCR	5' CATTCTACACCCCCAAGTGC 3'	5' CTGATAGGCGAAGGCAATGT 3'	mouse
	Vegfa qPCR	5' GGTTCCAGAAGGGAGAGGAG 3'	5' GGCAGTAGCTTCGCTGGTAG 3'	mouse
	Kcnt2 qPCR	5' AAGGCTGGCAAAATGATGAC 3'	5' CTGTGACGGTTTCTCAAGCA 3'	mouse
	Wnt7a qPCR	5' GGTGCGAGCATCATCTGTAA 3'	5' TGGTACTGGCCTTGCTTCTC 3'	mouse
sgRNA	*Thoc6*$^{fs/fs}$ sgRNA	5' CACCGCACCGCTCGCGGTGCCTCT 3'	5' CAGAGGCACCGCGAGCGGTGCCAAA 3'	mouse

Supplementary Data 1. RSEM counts for hNPCs, mouse E9.5 forebrain, and ERCC samples.

Supplementary Data 2. rMATS junction counts results for hNPCs. Results for *THOC6*$^{W100*/+}$ versus *THOC6*$^{E188K/E188K}$ highlighted in purple, and comparison of *THOC6*$^{W100*/+}$ versus *THOC6*$^{W100*/W100*}$ in green. Significance determined by likelihood-ratio test. Event types: SE, skipped exon; RI, retained intron; A5SS, alternative 5' splice site; A3SS, alternative 3' splice site; MXE, mutually exclusive exon. ΔPSI values are relative to affected genotypes. Inclusion level 1 is unaffected and inclusion level 2 is affected. Negative PSI indicates stronger inclusion in affected condition.

Supplementary Data 3. rMATS junction counts for mouse E9.5 forebrain. Results for comparison of *Thoc6*$^{+/+}$ versus *Thoc6*$^{fs/fs}$. Significance determined by likelihood-ratio test. Event types: SE, skipped exon; RI, retained intron; A5SS, alternative 5' splice site; A3SS, alternative 3' splice site; MXE, mutually exclusive exon. ΔPSI values are relative to *Thoc6*$^{fs/fs}$. Inclusion level 1 is for *Thoc6*$^{+/+}$ and inclusion level 2 is *Thoc6*$^{fs/fs}$. Negative PSI indicates stronger inclusion in affected condition.
