# Supplementary material for: TREX tetramer disruption alters RNA processing necessary for corticogenesis in THOC6 Intellectual Disability Syndrome
Source: Nat Commun. 2024 Feb 22;15:1640. doi: 10.1038/s41467-024-45948-y (PMC10884030; doi:10.1038/s41467-024-45948-y)
Supplement: Supplementary file 2 — Description of Additional Supplementary Files [file 41467_2024_45948_MOESM2_ESM.pdf]

Description of Additional Supplementary Files

File Name: Supplementary Data 1
Description: **RSEM counts for hNPCs, mouse E9.5 forebrain, and ERCC samples.**

File Name: Supplementary Data 2
Description: **rMATS junction counts results for hNPCs.** Results for $THOC6^{W100*/+}$ versus $THOC6^{E188K/E188K}$ highlighted in purple, and comparison of $THOC6^{W100*/+}$ versus $THOC6^{W100*/W100*}$ in green. Significance determined by likelihoodratio test. Event types: SE, skipped exon; RI, retained intron; A5SS, alternative 5’ splice site; A3SS, alternative 3’ splice site; MXE, mutually exclusive exon. ΔPSI values are relative to affected genotypes. Inclusion level 1 is unaffected and inclusion level 2 is affected. Negative PSI indicates stronger inclusion in affected condition.

File Name: Supplementary Data 3
Description: **rMATS junction counts for mouse E9.5 forebrain.** Results for comparison of $Thoc6^{+/+}$ versus $Thoc6^{fs/fs}$. Significance determined by likelihood-ratio test. Event types: SE, skipped exon; RI, retained intron; A5SS, alternative 5’ splice site; A3SS, alternative 3’ splice site; MXE, mutually exclusive exon. ΔPSI values are relative to $Thoc6^{fs/fs}$. Inclusion level 1 is for $Thoc6^{+/+}$ and inclusion level 2 is $Thoc6^{fs/fs}$. Negative PSI indicates stronger inclusion in affected condition.
