# Supplementary material for: TREX tetramer disruption alters RNA processing necessary for corticogenesis in THOC6 Intellectual Disability Syndrome
Source: Nat Commun. 2024 Feb 22;15:1640. doi: 10.1038/s41467-024-45948-y (PMC10884030; doi:10.1038/s41467-024-45948-y)
Supplement: Supplementary file 6 — Reporting Summary [file 41467_2024_45948_MOESM6_ESM.pdf]

nature portfolio

Corresponding author(s): Stephanie L. Bielas; Ashleigh Schaffer

Last updated by author(s): 12/13/2023

Reporting Summary

Nature Portfolio wishes to improve the reproducibility of the work that we publish. This form provides structure for consistency and transparency in reporting. For further information on Nature Portfolio policies, see our Editorial Policies and the Editorial Policy Checklist.

Statistics

For all statistical analyses, confirm that the following items are present in the figure legend, table legend, main text, or Methods section.

n/a	Confirmed	
☐	☒	The exact sample size (*n*) for each experimental group/condition, given as a discrete number and unit of measurement
☐	☒	A statement on whether measurements were taken from distinct samples or whether the same sample was measured repeatedly
☐	☒	The statistical test(s) used AND whether they are one- or two-sided<br>*Only common tests should be described solely by name; describe more complex techniques in the Methods section.*
☐	☒	A description of all covariates tested
☐	☒	A description of any assumptions or corrections, such as tests of normality and adjustment for multiple comparisons
☐	☒	A full description of the statistical parameters including central tendency (e.g. means) or other basic estimates (e.g. regression coefficient) AND variation (e.g. standard deviation) or associated estimates of uncertainty (e.g. confidence intervals)
☐	☒	For null hypothesis testing, the test statistic (e.g. *F*, *t*, *r*) with confidence intervals, effect sizes, degrees of freedom and *P* value noted<br>*Give P values as exact values whenever suitable.*
☒	☐	For Bayesian analysis, information on the choice of priors and Markov chain Monte Carlo settings
☒	☐	For hierarchical and complex designs, identification of the appropriate level for tests and full reporting of outcomes
☒	☐	Estimates of effect sizes (e.g. Cohen's *d*, Pearson's *r*), indicating how they were calculated

Our web collection on statistics for biologists contains articles on many of the points above.

Software and code

Policy information about availability of computer code

Data collection

This paper does not report original code. No software was used for data collection.

Data analysis

This paper does not report original code. Variant calling of single nucleotide variants (SNVs) and copy number variants (CNVs) was performed using GATKv4, VEP, and CoNIFERv0.2.2. Chromatograms were analyzed using Sequencer (v5.4.6) and Geneious Prime Software (v.2022.1.1). For RNAseq, FASTQ files were trimmed with Cutadapt v4.1 using default parameters. Read quality was assessed by FASTQC v0.11.9114. MultiQC v1.7115 was used to visualize FASTQC outputs and compare samples. FASTQ reads were then mapped to merged files using STAR alignment with parameter '--outSAMtype BAM SortedByCoordinate'. CNV microarray analysis on cell lines was performed with Genome Studio v2.0. Count analysis was performed on sorted BAM files using RSEM with paired-end alignment specified. Differential expression analysis was carried out using DESeq2 v1.34.0118 in R v4.1.2119. Volcano and PCA plots were made using ggplot2 and pcaExplorer packages in R. Alternative splicing analysis was performed on sorted BAM files using rMATS v4.1.2120 with the following parameters: '-t paired --readLength 150 --variable-read-length --nthread 4'. Events with less than 5 average reads were filtered out using the MASER package in R. To calculate splice site strength at 5' and 3' splice sites in AS transcripts identified by rMATS, maximum entropy modeling was carried out using MaxEntScan. Graphs were made in GraphPad Prism (v9.3.1). DAVID (david.ncifcrf.gov/tools) and Metascape (metascape.org) analyses were performed to identify enriched biological pathways based on Benjamini-Hochberg multiple hypothesis corrections of the p-values. To explore evidence for RNA-binding protein motifs at AS junctions, CentriMo Local Motif Enrichment Analysis was performed (MEME Suite 5.5.2). To identify potential transcription factors responsible for expression differences, Gene Set Enrichment Analysis (GSEA v4.2.3) against the MSigDB transcription factor motif gene set (c4.tftv7.5.1.symbols.gmt) and ChIP-X Enrichment Analysis v3 (ChEA3) were performed. Ensembl BioMart tool (http://useast.ensembl.org/biomart) was used to obtain coding sequence length, transcript number per gene, gene type, and sequences for AS events. The GeneOverlap v1.32 R package was used to identify overlapping DE and AS hits between affected genotypes. Primary and candidate syndromic ID genes were obtained from the SysID database (https://www.sysid.dbmr.unibe.ch). Imaging was performed with a Nikon A1ss inverted confocal microscope using NIS-Elements Advanced Research software. Image analysis was performed using Fiji

(ImageJ2v2.9.0) and CellProfiler (v4.2.1). Quantitative real-time PCR (qPCR) was performed using the Applied Biosystems 7500 system with 7500 Software v2.3. GeneOverlap, ggplot2, and pcaExplorer packages were used in R (v4.2.2).

For manuscripts utilizing custom algorithms or software that are central to the research but not yet described in published literature, software must be made available to editors and reviewers. We strongly encourage code deposition in a community repository (e.g. GitHub). See the Nature Portfolio guidelines for submitting code & software for further information.

Data

Policy information about availability of data

All manuscripts must include a data availability statement. This statement should provide the following information, where applicable:

- Accession codes, unique identifiers, or web links for publicly available datasets
- A description of any restrictions on data availability
- For clinical datasets or third party data, please ensure that the statement adheres to our policy

Source data are provided with this paper. The raw RNAseq data generated in this study have been deposited in the GEO database under accession code GSE245121 [https://www.ncbi.nlm.nih.gov/geo/query/acc.cgi?acc=GSM7837075]. The clinical data and the processed RNAseq data generated in this study are provided in the Supplementary Information/Source Data file. Genome references used include human GRCh387p13, human GRCh38.p13 reference, and mouse GRCm39. Databases and datasets used include 1000 Genomes Project phase 311, Genome Aggregate Database (gnomADv3), National Heart, Lung, and Blood Institute Exome Sequencing Project Exome Variant Server (ESP6500SI-V2), SysID database (https://www.sysid.dbmr.unibe.ch), and the MSigDB transcription factor motif gene set (c4.tftv7.5.1.symbols.gmt). Further information and requests for resources and reagents should be directed to and will be fulfilled by the lead contact, Stephanie L. Bielas (sbielas@umich.edu).

Research involving human participants, their data, or biological material

Policy information about studies with human participants or human data. See also policy information about sex, gender (identity/presentation), and sexual orientation and race, ethnicity and racism.

Reporting on sex and gender

Assigned sex at birth for Probands 1, 3, 6, and 7 was male. Assigned sex at birth for Probands 2, 4.1, 4.2, and 5 was female. Sex-influenced phenotypes relevant to the TIDS clinical report include genitourinary defects. No further sex- or gender-based analysis were performed given that this manuscript is focused on neurodevelopmental defects which are not influenced by sex/gender.

Reporting on race, ethnicity, or other socially relevant groupings

Probands 1 and 3 are of European ancestry; Proband 2 is of Turkish ancestry; Probands 4.1 and 4.2 are of Southern Indian ancestry; Proband 5 is of Moroccan ancestry.

Population characteristics

P1 was from the Netherlands of European ancestry. Age at last examination was 12 months old and genotype was compound heterozygous for THOC6:c.569G>A,p.G190E; c.139C>T, p.Q47*. Karyotyping results were normal. Diagnosed with TIDS.

P2 was of Turkish ancestry born from consanguineous parents. Age at last examination was 3 years old and genotype was homozygous for THOC6:c.299G>A,p.W100*. Karyotyping results were normal. Diagnosed with TIDS.

P3 was from the USA of European ancestry. Age at last examination was 11 years old and genotype was homozygous for THOC6:c.[298T>A;700G>C;824G>A], (p.[W100R;V234L;G275D]). Karyotype and FISH analysis for 22q11.2 were normal (Oxford Gene Technology Syndrome). Chromosome microarray (Affymetrix CytoScan Dx) identified a maternally inherited deletion on 5q21.1 inconsistent with phenotype. Diagnosed with TIDS.

P4.1 and P4.2 are siblings of third-degree consanguineous parents from Southern India. Age at last examination for P4.1 and P4.2 were 13 years old and 8 years old, respectively. Genotypes for P4.1 and P4.2 were homozygous for THOC6:c.824G>A,p.G275D. Karyotyping results were normal for P4.1 and P4.2. Both were diagnosed with TIDS.

P5 was of Moroccan ancestry born from consanguineous parents. Age at last examination was 10 years old. Genotype was homozygous for THOC6:c.740G>A,p.R247Q. 800-bands resolution karyotype showed normal chromosomes on lymphocytes, 46XX, with no 22q11.2 deletion by FISH analysis at the TUPPLE1 locus. Diagnosed with TIDS.

P6 was 13 months old at last examination. Ancestry was not reported. Genotype was homozygous for THOC6:c.562G>A,p.E188K. Diagnosed with TIDS.

P7 was homozygous for THOC6:c.299G>A,p.W100*. Ancestry and age at last examination were not reported. Diagnosed with TIDS.

Recruitment

Our inclusion criteria for this study was diagnosis of TIDS due biallelic pathogenic variants in THOC6. Selection bias is due to clinicians and families willing to participate in research. Probands 4.1 and 4.2 were recruited at Kasturba Medical College, Manipal, Manipal Academy of Higher Education, Manipal, 576104, India. Probands 6-7 were affiliated with Case Western Reserve University. Probands 1-3 and 5 were identified through GeneMatcher.

Ethics oversight

All participants or parents/guardians in this study were consented under an approved institutional review board. In all cases, the procedures followed were in accordance with the ethical standards of the respective institution’s committee on human research (Radboud University Medical Centre Nijmegen, The Netherlands (Family (F) 1); Marmara University Hospital Pediatric Allergy and Immunology, Istanbul, Turkey (F2); Greenwood Genetic Center, Greenwood, South Carolina, USA (F3); Kasturba Medical College, Manipal, India (F4); Imagine Institute, Paris, France (F5); Case Western Reserve University, OH, USA (F6-F7)) and were in keeping with international standards. Consent for publishing individual-level data was provided by all

participants and/or parents/guardians. The authors affirm that all human research participants [or their parents/guardians] provided informed consent for publication of the images in Figure 1.

Note that full information on the approval of the study protocol must also be provided in the manuscript.

Field-specific reporting

Please select the one below that is the best fit for your research. If you are not sure, read the appropriate sections before making your selection.

☒ Life sciences ☐ Behavioural & social sciences ☐ Ecological, evolutionary & environmental sciences

For a reference copy of the document with all sections, see nature.com/documents/nr-reporting-summary-flat.pdf

Life sciences study design

All studies must disclose on these points even when the disclosure is negative.

Sample size	No sample-size calculations were performed. Experiment was designed based on genotypes. For human iPSCs, five genotypes were used for analyses based on the patient lines we were able to obtain (wildtype, heterozygous, and homozygous affected; both missense and nonsense variants were represented). All downstream experiments were performed on a minimum n = 3 biological replicates. Given that THOC6 is necessary for early neural development, the cortical differentiation of these lines is difficult, and served as a limiting factor for differentiation replicate size. For RNAseq analysis, a total of n=4 biological differentiation replicates per condition were achieved (affected or unaffected) (with n = 2 per variant). To confirm initial discovery experiments findings from RNAseq on n = 4 biological replicates per condition (n = 2 biological replicates per genotype), all downstream validation experiments were performed in 3 additional, independent differentiation replicates. All organoid experiments were done in triplicate.<br><br>For mouse embryos, three genotypes were used based on editing strategy for hypothesis testing (wildtype, heterozygous, and homozygous frameshift). Mouse embryo sample sizes were chosen based upon the phenotypic effect size and variance measured between Thoc6fs/fs mutant embryos and control for each reported experiment. The minimum number of mice required to obtain significance and enable statistical analysis was used in accordance with institutionally-approved IACUC protocols.
Data exclusions	E188K/+ NPCs biological replicates were excluded from main text RNAseq experiments based on the following reasons: PCA analysis determined it was an outlier and this was consistent with the differential expression analysis, which detected an abundance of upregulated skeletal muscle genes in independent THOC6E188K/+ hNPC differentiation replicates, indicating unanticipated issues during the differentiation of this cell line. Variability in differentiation capacity and cellular composition following differentiation is a commonly observed issue when working with iPSCs. However, RNAseq analyses using E188K/+ NPCs, which are provided in supplemental data, support the same trend on RNA processing observed when excluding it, giving us confidence in the described molecular phenotypes.
Replication	THOC5 co-IP experiments were repeated three times independently and showed similar results (Figures 2F and S1H).<br>Steady-state protein Western blot experiments were repeated three times independently and showed similar results (Figure 1G)<br>Ataluren experiment was repeated twice with similar results observed (Figure 1G).<br>RT-qPCR validation of RNAseq hits was repeated twice independently and showed similar results (Figure 4F and S7G)<br>ActD RNA stability experiment was repeated three times independently and similar results were obtained (Figures 1F, S1C-E).<br>Allele-specific RT-PCR experiment confirming alternative splicing events was performed twice with similar results (Figures S3G and S7B).<br>Western blot experiment for protein confirmation of RNAseq DE hits was performed once (Figure 4H).<br>Oligo-dT FISH experiments to assess export defects was repeated three times independently with similar results (Figures S2 and S6F).<br>Organoid and NPC differentiations were repeated three and five independent times, respectively, and showed similar results
Randomization	Trial groups were separated based on genotype. Human pluripotent stem cell models were differentiated under the same culture conditions and when indicated, embryoid bodies were split for either organoid generation or NPC generation solely based on number of organoids formed per batch. Randomized generation of neuroepithelial rosette structures within control and affected organoids were ensured through established in vitro differentiation protocols using 96-well V-shaped plates across several replicates. All genotypes for mouse litters were used and thus trial groups were defined by genotypes within a litter. There are very few variables to incorporate in our groups are randomized for our experimental design.
Blinding	All computational and quantification analyses were performed blinded to genotype of samples.

Reporting for specific materials, systems and methods

We require information from authors about some types of materials, experimental systems and methods used in many studies. Here, indicate whether each material, system or method listed is relevant to your study. If you are not sure if a list item applies to your research, read the appropriate section before selecting a response.

Materials & experimental systems

n/a	Involved in the study
☐	☒ Antibodies
☐	☒ Eukaryotic cell lines
☒	☐ Palaeontology and archaeology
☐	☒ Animals and other organisms
☒	☐ Clinical data
☒	☐ Dual use research of concern
☒	☐ Plants

Methods

n/a	Involved in the study
☒	☐ ChIP-seq
☒	☐ Flow cytometry
☒	☐ MRI-based neuroimaging

Antibodies

Antibodies used

Primary antibodies used for Western blot analysis: mouse anti-THOC6 (1:1000, Abnova H00079228-A01, polyclonal), rabbit anti-THOC1 (1:2000, Bethyl Laboratories A302-839A, polyclonal), rabbit anti-THOC2 (1:2000, Bethyl Laboratories A303-630A, polyclonal), rabbit anti-THOC5 (1:2000, Bethyl Laboratories A302-120A, polyclonal), mouse anti-ALYREF (1:2000, Sigma Aldrich A9979, monoclonal), rabbit anti-CHTOP (1:2000, Invitrogen PA5-55929, polyclonal), mouse anti-β-actin (1:5000, Abcam ab6276, monoclonal), goat anti-HAPLN1 (1:400, R&D Systems AF2608, polyclonal), rabbit anti-CEMIP (1:2500, Proteintech 50-173-3270, polyclonal), rabbit anti-WNT7A (1:1000, Abcam ab274321, monoclonal), rabbit anti-DKK2 (1:250,ab38594, polyclonal), rabbit anti-TP53 (1:1000, Abcam ab131442, polyclonal), rabbit anti-Flag antibody (1:2500, Proteintech 20543-1-AP) . Secondary antibodies used: donkey anti-rabbit HRP-conjugated (1:5000, Cytiva NA9340V), goat anti-mouse HRP-conjugated (1:1000, Invitrogen 32430), IRDye® 680RD goat anti-rabbit IgG (1:5000, Li-Cor Biosciences 926-68071), and IRDye® 800CW goat anti-mouse IgG (1:5000, Li-Cor Biosciences 925-32210).

Primary antibodies used for IHC/ICC: rabbit anti-PAX6 (1:100, BioLegend PRB-278P, polyclonal), rabbit anti-KI67 recombinant (1:200, Abcam ab16667), rat anti-PH3 (1:250, Abcam ab10543, monoclonal), rabbit anti-cleaved caspase-3 (1:100-1:400, Cell Signaling 9661, polyclonal), mouse anti-N-Cadherin (BD Biosciences 610920, monoclonal), goat anti-DCX (1:400, Santa Cruz Biotechnology C-18, sc-8066, polyclonal), rat anti-CTIP2 (1:500, Abcam ab18465, monoclonal), and goat anti-SOX1 (1:100, R&D Biosystems AF3369, polyclonal). AlexaFluor-conjugated secondaries used: donkey anti-mouse 647 (1:400, Invitrogen A31571), donkey anti-rat 555 (1:400, Invitrogen A48270), and donkey anti-rabbit 488 (1:400, Invitrogen A21206).

Validation

All antibodies used were commercial and used according to the manufacturer's instructions. Concentrations were optimized in our lab and based on previous citations.

mouse polyclonal anti-THOC6 [1:1000, Abnova H00079228-A01); used successfully in this study in human and mouse cells and validated with THOC6 overexpression plasmid in HEK293-Ts – Figures 1G, 2F, 6C, and S2A); WB application].

rabbit polyclonal anti-THOC1 [1:1000 dilution for WB, 1:250 dilution for ICC; based on PMID: 30476144 (Figures 2E-F, S1F); Bethyl Laboratories A302-839A, validation statement provided ("All Bethyl Laboratories® antibodies are validated to meet our strict performance standards."); previous citations of this antibody (PMID: 32116545); used in this study successfully in WB and ICC applications in human cells].

rabbit polyclonal anti-THOC2 (1:2000 dilution used in this study and based on PMID: 30476144); Bethyl Laboratories A303-630A, validation statement provided ("All Bethyl Laboratories® antibodies are validated to meet our strict performance standards."); previous citations of this antibody (PMID: 32116545); present on CiteAb database; used in this study successfully in WB applications in human cells].

rabbit polyclonal anti-THOC5 [1:2000 dilution used in this study; based on PMID: 32116545 (Figure); Bethyl Laboratories A302-120A, validation statement provided ("All Bethyl Laboratories® antibodies are validated to meet our strict performance standards."); previous citations of this antibody (PMID: 36590164, 33483601, 32116545, 30476144, 21937706); present on CiteAb database for WB application; used in this study successfully in WB applications in human cells].

mouse monoclonal anti-ALYREF [1:2000 dilution for WB, 1:250 dilution for ICC, Sigma Aldrich A9979; clone 11G5, purified from hybridoma cell culture; 14 previous citations of this antibody (e.g., PMID: 32854341, 37679383, 35704758); present on CiteAb database for WB/ICC/IF applications; used in this study successfully in WB and ICC applications in human cells in human cells].

rabbit polyclonal anti-CHTOP (1:2000 dilution for WB, 1:250 dilution for ICC; Invitrogen PA5-55929; tested in WB, IHC, ICC/IF applications by manufacturer; used successfully in this study for WB and ICC applications in human cells].

Mouse monoclonal anti-β-actin (1:5,000, Abcam ab6276); manufacturer statement (Abpromise guaranteed for use with WB and ICC/IF applications with 76 five-star reviews for WB; knockout validated); on CiteAb database for WB, ICC/IF, IHC, and more applications; 2,565 citations for this product in human and mouse cells]

goat polyclonal anti-HAPLN1 [1:400, R&D Systems AF2608, specificity statement ("Detects human HAPLN1 in direct ELISAs and Western blots. In these formats, this antibody shows approximately 5% cross¬reactivity with recombinant human HAPLN4."); tested in WB and ELISA application by manufacturer; 13 previous citations of this antibody (e.g., PMID: 36102038, 35454094, 34604231); present on CiteAb database for IHC, WB, ICC, IF applications; used in this study successfully in WB applications in human cells].

Rabbit polyclonal anti-CEMIP [1:500, Proteintech 21129-1-AP; validation statement 'KD/KO Validated'; tested in ICC/IF, IHC, and WB applications by manufacturer; 42 previous citations of this antibody; present on CiteAb database for WB/IHC/IF application; used in

this study successfully in WB applications in human cells].

rabbit monoclonal anti-WNT7A [1:1000, Abcam ab274321; manufacturer's statement (Abpromise guaranteed for use with WB and ICC/IF applications); tested in ICC/IF and WB applications by manufacturer; 3 previous citations of this antibody (PMID: 36646807, 34647904); present on CiteAb database for WB application; used in this study successfully in WB applications in human cells].

rabbit polyclonal anti-DKK2 [1:250, Abcam ab38594; manufacturer's statement (Abpromise guaranteed for use with WB and IHC applications; 9 previous citations of this antibody (PMID: 36747258, 28467796, 31783893); present on CiteAb database for WB, IHC, ICC/IF applications; used in this study successfully in WB applications in human cells].

Rabbit polyclonal anti-TP53 [1:1000, Abcam ab131442; manufacturer's statement (KO validated; Abpromise guaranteed for use with WB and IHC applications); 287 previous citations of this antibody (e.g., PMID: 37450438, 36496404); present on CiteAb database for WB, ICC, IHC, ChIP applications; used in this study successfully in WB applications in human cells].

rabbit anti-Flag polyclonal [1:2500, Proteintech 20543-1-AP validation statement (validated in WB); 771 previous citations of this antibody; present on CiteAb database for WB, IP, IF, ChIP and more applications; used in this study successfully in WB applications in human cells].

rabbit polyclonal anti-PAX6 [1:100, BioLegend PRB-278P; manufacturer's statement ("Each lot of this antibody is quality control tested by Western blotting and formalin-fixed paraffin-embedded immunohistochemical staining of brain tissue."); 337 previous citations of this antibody; used in this study successfully in IHC applications in human cells].

rabbit anti-KI67 recombinant [1:200, Abcam ab16667; validation statement (knockout validated; Abpromise guaranteed for use with WB, ICC, and IHC applications); 3,115 previous citations of this antibody; present on CiteAb database for IHC, ICC, IF, WB, and more applications; used in this study successfully in IHC applications in human cells].

rat anti-PH3 monoclonal [1:250, Abcam ab10543; manufacturer's statement (Abpromise guaranteed for use with WB, ICC, and IHC applications); 147 previous citations of this antibody; present on CiteAb database for IHC, IF, WB, ICC, applications; used in this study successfully in IHC applications in human and mouse tissue].

rabbit anti-cleaved caspase-3 polyclonal [1:100-1:400, Cell Signaling 9661; 9,661 previous citations of this antibody; present on CiteAb database for WB, IHC, IF, ICC applications; used in this study successfully in IHC applications in human and mouse tissue].

mouse anti-N-Cadherin monoclonal [BD Biosciences 610920; manufacturer's statement (applications: Western blot (Routinely Tested), Immunofluorescence (Tested During Development), Immunoprecipitation (Reported)); 560 previous citations of this antibody; present on CiteAb database for WB, IHC, IF, ICC application; used in this study successfully in IHC applications in human cells].

goat anti-DCX polyclonal [1:400, Santa Cruz Biotechnology, C-18, sc-8066; 225 previous citations of this antibody; used in this study successfully in IHC applications in human cells].

rat anti-CTIP2 monoclonal [1:500, Abcam ab18465; manufacturer's statement (Abpromise guaranteed for use with WB, ICC, and IHC applications); 986 previous citations of this antibody; present on CiteAb database for IHC, ICC, WB, and more applications; used in this study successfully in IHC applications in human cells].

goat anti-SOX1 polyclonal [1:100, R&D Biosystems AF3369; 165 previous citations of this antibody; present on CiteAb database for ICC, IHC application; used in this study successfully in IHC applications in human cells].

Eukaryotic cell lines

Policy information about cell lines and Sex and Gender in Research

Cell line source(s)	Commercial lines used in this manuscript include: H9 (THOC6+/+, 46XX, ESCs, WA09, WiCell).<br><br>The following lines used in this manuscript were established as part of the Diabetes iPSC Panel by the New York Stem Cell Foundation (NYSCF) and is available to researchers through the biorepository at NYSCF (nyscf.org/research-institute/repository-stem-cell-search/): AS0035 (THOC6+/+, 46XX, iPSCs, NYSCF Diabetes iPSC Panel), and AS0041 (THOC6+/+, 46XY, iPSCs, NYSCF Diabetes iPSC Panel).<br><br>The following iPSC lines were reprogrammed from primary skin fibroblasts: KMC6002 [THOC6E188K/+, 46XY (assigned male at birth)], KMC6003 [THOC6E188K/E188K, 46XY (assigned male at birth)], KMC7001 [THOC6W100*/+, 46XY (assigned male at birth)], and KMC7002 [THOC6W100*/W100*, 46XY (assigned male at birth)]. Lines KMC6002 and KMC6003 were reprogrammed from primary skin fibroblasts obtained from individual P6 and the unaffected father. Lines KMC7001 and KMC7002 were reprogrammed from primary skin fibroblasts obtained from individual P7 and the unaffected father.
Authentication	Sanger sequencing validation of genotypes and CNV microarray analysis (Illumina Bead Array, analysis with Genome Studio v2.0) were performed on all lines to ensure no pathogenic changes were acquired during culturing. No further validation of iPSCs lines was performed in our lab.
Mycoplasma contamination	All cell lines were routinely tested negative for mycoplasma contamination.
Commonly misidentified lines (See ICLAC register)	No commonly misidentified lines were used in this study.

Animals and other research organisms

Policy information about studies involving animals; ARRIVE guidelines recommended for reporting animal research, and Sex and Gender in Research

Laboratory animals	C57BL/6JN hybrid mice (Jackson Laboratory, 005304) were used for CRISPR editing of the Thoc6 locus. Founder mice with the Thoc6fs/+ allele were intercrossed with C57BL/6JN mice (Jackson Laboratory, 005304) for line maintenance. All ex vivo analyses were performed on tissue collected from mice of both sexes at embryonic day (E) 8.5-10.5. Sex-dependent differences were not assessed. The animals described in this study were housed in the AAALAC accredited facilities of the CWRU School of Medicine (SOM). Standard Operating Procedures and reference materials are available from the IACUC Office for animal use.
Wild animals	No wild animals were used in the study.
Reporting on sex	All ex vivo analyses were performed on tissue collected from mice of both sexes at embryonic day (E) 8.5-10.5. Sex-dependent differences were not assessed.
Field-collected samples	No field-collected samples were used in the study.
Ethics oversight	All mice were maintained according with the National Institutes of Health Guidelines for the Care and Use of Laboratory Animals and were approved by the Case Western Reserve Institutional Animal Care and Use Committee.

Note that full information on the approval of the study protocol must also be provided in the manuscript.
